# The pQBR mercury resistance plasmids: a model set of sympatric environmental mobile genetic elements

**DOI:** 10.1099/mgen.0.001789

**Published:** 2026-07-22

**Authors:** Victoria T. Orr, Ellie Harrison, Damian W. Rivett, Rosanna C. T. Wright, James P. J. Hall

**Affiliations:** 1 Department of Evolution, Ecology and Behaviour, Institute of Infection, Veterinary and Ecological Sciences, University of Liverpool, Liverpool, UK; 2 School of Biosciences, University of Sheffield, Sheffield, UK; 3 Department of Natural Science, Manchester Metropolitan University, Manchester, UK; 4 Division of Evolution, Infection and Genomics, University of Manchester, Manchester, UK

**Keywords:** antibiotic resistance, megaplasmid, plasmid, Pseudomonas, transposon, soil microbiology

## Abstract

Plasmids are extrachromosomal mobile genetic elements that can facilitate rapid bacterial adaptation by transferring genes between individuals. Whilst plasmids are known to exist in diverse habitats and encode a range of traits, most of our knowledge about plasmids comes from clinically associated antimicrobial resistance (AMR) plasmids that have already been recruited as vectors of drug resistance and have likely been shaped by strong selection for plasmid-encoded antibiotic resistance. Here, we investigated 26 plasmids from the pQBR collection – a set of large, co-existing mercury resistance environmental plasmids isolated in *Pseudomonas* spp. from a field in Oxfordshire in the 1990s – and explored the ability of pQBR plasmids to transfer novel chromosomally encoded traits. New whole-genome sequences for 25 plasmids confirmed that these soil-isolated plasmids are generally very large (140–588 kb), constitute at least six distinct genetic groups and have relatives in various other *Pseudomonas* species and habitats. Despite significant nucleotide-level divergence, Groups I (pQBR103-like, ~406 kb) and IV (pQBR57-like, ~328 kb) showed remarkable ancient similarities in synteny and gene content both with one another and with the PInc-2/IncP-2 family of plasmids known to transfer clinically significant drug resistance between *Pseudomonas aeruginosa* hosts. None of the pQBR plasmids sequenced to date harboured known AMR determinants, but putative phage defence systems and metal resistances were evident. Transposable elements, including the Tn*5042* mercury resistance transposon, were responsible for significant structural variation within plasmid groups, consistent with a predominant role of transposons in rapidly remodelling plasmids. To experimentally test the ability of pQBR plasmids to spread new traits, we developed a novel transposon transfer assay which showed that certain Group IV pQBR plasmids were especially effective at acquiring the chromosomally encoded transposon Tn*6291* and that this ability to transfer transposons was likely due to specific plasmid factors rather than generic conjugation rate. Our work presents a tractable set of sequenced plasmids suitable for exploring the evolution and dynamics of gene acquisition by pre-AMR plasmids and provides a key case study highlighting the pervasive interplay between plasmids and transposable elements that can drive microbial genome evolution.

**Impact Statement**

Plasmids can drive microbial evolution by acting as vectors for horizontal gene transfer. Because of their central role in disseminating antimicrobial resistance (AMR), plasmids are mainly explored as vehicles for AMR traits, meaning that our knowledge of the diversity and evolutionary dynamics of non-AMR plasmids is more limited. Here, we explore sequences from a set of mercury resistance plasmids that lack AMR determinants, isolated from pristine agricultural land in *Pseudomonas* spp. By providing new whole-genome sequencing analyses, we expand the set of sequenced pQBR plasmids to 26, finding globally dispersed relatives from clinical, environmental and industrial settings, and identifying an ancient plasmid backbone shared amongst divergent modern environmental and clinical AMR plasmids. We experimentally verify the role of pQBR plasmids in readily transferring chromosomal traits to a new host using a novel transposon transfer assay, which suggests that specific plasmid-transposon interactions may drive trait spread. Overall, our work expands our understanding of the role of environmental plasmids in acquiring and disseminating adaptive traits.

## Data Summary

The authors confirm that all supporting data, code and protocols have been provided within the article, through supplementary data files, or a GitHub repository https://github.com/jpjh/PQBR_PLASMIDS on Zenodo at https://doi.org/10.5281/zenodo.21210847. Sequence data have been deposited in the European Nucleotide Archive under project PRJEB71352. Accession numbers are provided in Supplementary Table S1.

## Introduction

Plasmids are mobile genetic elements (MGEs) that exist separately from the chromosome as (usually) circular pieces of DNA and can accelerate bacterial adaptation by transferring genes between individuals, predominantly via conjugation [1,2]. Plasmid genomes can be conceptually divided into conserved ‘backbone’ sections that usually harbour genes directly responsible for plasmid fitness – such as those encoding machinery for plasmid replication and transmission – and a diverse and flexible accessory genome that can vary between otherwise closely related plasmids and often encodes traits that affect the fitness of host bacteria, with indirect consequences for plasmid fitness [3]. Accessory genes can provide a plethora of functions, including resistance to antibiotics, biocides and metals; exotic metabolic and biodegradative pathways; and defence systems against other MGEs, including bacteriophage or even competing plasmids [4,8]. Plasmid-mediated gene transfer poses a grave threat in the context of antimicrobial resistance (AMR) in pathogens [9,11] but also offers opportunities for bioremediating polluted sites [12] or introducing novel traits into microbiomes [13,14], indicating the value of understanding the biological diversity of environmental conjugative plasmids.

Transposons [also known as transposable elements (TEs)] are regions of DNA encoding enzymes, called transposases, that interact with flanking DNA regions to catalyse the integration of the transposon in a different site [2]. Transposon activity can result in the proliferation of a transposon within a genome and enable the transfer of the transposon into new genomes, such as in cases where the transposon inserts into another MGE, e.g. a conjugative plasmid. Here, the plasmid acts as a ‘vehicle’, enabling the transposon to escape the confines of a single bacterial lineage and transfer via the plasmid into new backgrounds. Besides transposases, transposons can carry a cargo of accessory genes – in fact, many genes of interest within plasmid genomes, such as those conferring resistance traits, tend to be encoded on transposons [11,15]. These interactions between plasmids and transposons can accelerate the spread of traits, as has been observed for resistance genes in hospital settings [16,17], because transposons enable genes to switch between plasmids, as well as on and off chromosomes [18]. When comparing plasmid sequences across isolates, these patterns manifest as a conserved backbone region alongside ‘hotspot’ regions of great genetic diversity, as was observed for the IncP-1 plasmids [19].

Much of what we know about plasmids comes from antibiotic resistance plasmids isolated from clinical settings in the years following the widespread introduction of antibiotics in healthcare and agriculture. Strong selection for resistance can shape plasmid dynamics and evolution in various ways. For example, by driving high plasmid frequency in a population, selection can reduce opportunities for plasmid-mediated gene transfer [20] and favour mutations that increase copy number [21,22]. Acquisition of resistance genes can also alter plasmid fitness effects [20,23]. Comparative studies that span the pre- and post-antibiotic eras show that modern-day resistance plasmids have been recruited from a wider plasmid pool and are closely related to historic plasmid backbones lacking resistance genes [24]. Developing a broader understanding of plasmid dynamics and evolution therefore requires study of plasmids that have not undergone strong selection for specific resistance traits.

The pQBR collection is a set of sympatric plasmids isolated from the phytosphere of sugar beets grown at Oxford University Farm, Wytham, Oxford, UK [25,27]. These plasmids were captured using exogenous isolation, whereby a rifampicin-resistant *Pseudomonas putida* UWC1 recipient [28] or a kanamycin-resistant *Pseudomonas fluorescens* SBW25 recipient [29] was allowed to grow alongside the sugar beet microbiome before selection using rifampicin/kanamycin and mercuric chloride (HgCl_2_). The experimental design means that the original host of these plasmids in the sugar beet phytosphere is unknown, but at the moment of capture, they were evidently mobilizable, able to replicate in *Pseudomonas* species and confer phenotypic mercury resistance. An abundance of pQBR plasmids was captured this way – despite the source site having insufficient levels of mercury for positive selection and no known history of mercury pollution [26,27] – and was classified into groups based on restriction fragment length pattern (RFLP) similarities [25].

Experimental work performed on the pQBR plasmids over the last 30 years has significantly extended our knowledge of plasmid evolutionary ecology. This includes work on plasmid associations with plant roots [26] and biofilms [30], plasmid fitness [31], transfer [32], stability [33,34] and compensatory evolution [35,38]. Sequences of pQBR103 [39], pQBR57, pQBR55 and pQBR44 [40], resolved in previous studies, revealed uncharacterized genetic novelty and hinted at vast unexplored diversity in the rhizosphere. There have also been studies focusing on particular groups of genes, such as plant-inducible helicases in pQBR103 [41] and origins of replication in pQBR11 and pQBR55 [42,43]. However, previous studies focused either on specific genes or on individual divergent representatives of the restriction pattern groupings defined by Lilley *et al*. [25]. As a result, we lack a detailed understanding of diversity and evolution within and between more closely related plasmids. Experimental studies have suggested the potential for rapid divergence between related pQBR plasmids by transposon acquisition and rearrangements [20], suggesting that these plasmids might be efficient vehicles for acquiring and transferring chromosomally encoded traits to other members of the soil microbiome.

Here, we increase the repertoire of sequenced pQBR plasmids, providing a snapshot of a community of co-existing plasmids in soil that have not been recruited for AMR gene carriage. We show that, consistent with earlier RFLP typing, the pQBR plasmids fall into distinct groups, with within-group structural variation driven by recent, prolific transposon insertions. The backbones of each group include numerous genes and operons with predicted activities extending beyond the replication and conjugation of the plasmid, including chemotaxis, radical S-adenosyl-l-methionine (SAM) metabolism and type IV pilus formation. Using a novel assay, we show that the pQBR plasmids vary widely in their capacity for transferring chromosomal transposons to a new host.

## Methods

### Bacterial culture

King’s B media (KB) was used to culture micro-organisms. KB broth was made with 20 g Bacto-Proteose peptone No. 3 (Difco), 1.5 g magnesium phosphate heptahydrate, 1.15 g potassium phosphate dibasic anhydrous and 10 g glycerol (Honeywell) per litre. Agar (12 g l^−1^) was added to make KB agar. All broth culturing, unless otherwise stated, was in 5 ml broth in 50 ml polypropylene tubes (Greiner Bio-One, 227261) within an Innova42 shaking incubator (New Brunswick Scientific) at 180 r.p.m. and 28 °C. Media supplementations with kanamycin were at 50 µg ml^−1^, mercuric chloride at 20 µM, streptomycin at 100 µg ml^−1^ and 5-bromo-4-chloro-3-indolyl-*β*-d-galactopyranoside (X-gal) at 50 µg ml^−1^.

### Bacterial strains

Original pQBR plasmid-carrying hosts were either *P. putida* UWC1 (a derivative of *P. putida* KT2440) or *P. fluorescens* SBW25, but prior to this study, all plasmids were transferred into *P. putida* UWC1 by conjugation as previously described [25,31]. Streptomycin-resistant (SmR) *P. fluorescens* SBW25 with a *lacZ* marker was previously described by Hall *et al*. [40]. *P. fluorescens* SBW25, harbouring a kanamycin resistance marker in Tn*6291* (SBW25-Tn*6291*::KmR), was produced from a *P. fluorescens* SBW25 wild-type [29] by homologous recombination [44] (full details in Supplementary Methods). *Pseudomonas koreensis* P19E3 plasmid p1, referred to here as pP19E3.1 [45], was previously transferred into *P. fluorescens* SBW25 [11].

### Antibiotic resistance analysis

AMR profiles were established by comparing plasmid-free and plasmid-carrying *P. putida* UWC1. Overnight cultures were spread (~1×10^7^ c.f.u. ml^−1^) onto Mueller–Hinton agar (with and without 0.1 µg ml^−1^ HgCl_2_) and tested using the M26/NCE multiple antimicrobial susceptibility ring (MAST Group Ltd.). Antimicrobials to which the *P. putida* UWC1 host was resistant were removed from the analysis, along with naladixic acid to remove possibilities of spontaneous mutations, leaving colistin, kanamycin, streptomycin and tetracycline as the test compounds. Zone of inhibition diameters were measured using digital callipers.

### Sequencing, assembly and bioinformatic analysis of pQBR plasmids

Initial whole-genome sequencing of the pQBR plasmid collection [25,27] was performed on *P. putida* UWC1 pQBR strains using Illumina [2×250 bp, minimum 30× coverage, Illumina sequencing performed by MicrobesNG (https://www.microbesng.com/)]. To identify plasmid sequences, reads were first mapped against reference *Pseudomonas* chromosomes using ‘bwa-mem’ [46], and reads that did not map to the chromosome were extracted using SAMtools [47]. This subset of reads was assembled using SPAdes v3.15.5 [48]. Contigs that had long (>5 kb) and deep coverage (>10× coverage) were identified as putative pQBR plasmid contigs and used for initial comparative analysis. These sequences were first compared with whole-genome SPAdes assemblies in Bandage [49] to identify and resolve plasmid sequences. To attempt to close the remaining sequences, we used Oxford Nanopore Technology (ONT) sequencing. In some cases, ONT sequencing was performed on pools of DNA extracted from cells with different plasmids; combinations were selected so similar plasmids were not present in the same sample. DNA was isolated using the Masterpure^™^ Complete DNA and RNA Purification Kit (Lucigen MC89010), and ONT bacterial genome sequencing was performed by Plasmidsaurus. ONT Flye (2.9.1-b1780) assemblies, polished with Medaka (1.8.0), were polished again using the corresponding Illumina reads with PolyPolish 0.5.0 [50,51], and direct repeats at the start and end of all assemblies were identified by ccfind 1.4.5 [52] and removed as assembly artefacts. These approaches produced closed plasmid sequences for 20 out of 28 UWC1(pQBR) strains; full details of the methods used to resolve all sequences are provided in Supplementary Text.

Group I, III and IV sequences were oriented using EMBOSS to place the first base of the first codon of a putative replication initiation protein (RIP) at the first position on the forward strand. Putative RIP genes were identified by querying a blast database from previously sequenced pQBR plasmids, with candidates chosen to facilitate visualization of synteny where multiple candidates were available. Group II plasmids were oriented relative to each other, with the first base set to be the first base of the first codon of a putative RIP. Plasmid pQBR26 was orientated similarly, based on the presence of a RepB family plasmid replication initiator protein gene. Initial analyses could not identify a putative RIP in pQBR105, so the sequence was not reoriented prior to annotation. Sequences, including those of the previously sequenced plasmids pQBR103 (AM235768.1), pQBR57 (LN713926.1), pQBR55 (LN713927.1) and pQBR44 (CDLQ010000001‐CDLQ010000002) [40], were annotated using bakta (v1.8.2, full database v.5.0.0) [53] on metagenome mode. Throughout, we use this (re-)annotation when referring to gene annotations and locus tags. Example analysis scripts and re-annotated plasmids are provided on GitHub (https://github.com/jpjh/PQBR_PLASMIDS).

Heatmaps were produced in R [54,56] using Mash distance [57] to cluster the sequences by similarity. Genome comparison plots were produced using tblastx and blastn, for intergroup and intragroup comparison, respectively [58], and plotted using EasyFig [59]. Sequences were uploaded to PHASTEST (v3.0) [60], TAFinder 2.0 (v3.0) [61] and DefenseFinder, including anti-defence finder (v2.2.0, Model 2.0.2) [62,63] and AMRFinderPlus (v4.0.23) [64], to identify potential phage insertions, putative toxin–antitoxin systems, putative genome defence and anti-defence systems and predicted AMR genes, respectively. Plasmid sequences were compared to the TnCentral database to identify any complete additional transposons present in the sequences [65]. MOBsuite was used to identify relaxases and examine predicted mobility [66]. Relatives were identified by querying PLSDB (v2023_11_23_v2) [67] and the complete genomes of PseudomonasDB (v22.1) [68] using Mash (sketch size 10,000) [57] with representatives from each group, and extracting sequences with an e-value <1e-100. Candidates were subsequently filtered for matches with at least one blastn match >5 kb and e<1e-40. Pangenomes were calculated using PIRATE [69] (v1.0.5), and nucleotide diversity for each gene cluster was calculated using Mega (v11.0.13). Phylogenetic trees of plasmids and relatives were constructed in R using PIRATE core genome alignment, trimAL (v1.5.rev0) and IQtree3 (v3.0.1) [54,70,74].

### Measuring and calculating conjugation and Tn*6291* transfer rates

Plasmids were transferred into SBW25-Tn*6291*::KmR by conjugation. KB broth (5 ml) was inoculated with 50 µl of a 50 : 50 v/v mix of overnight cultures of a *P. putida* UWC1 plasmid donor strain and the recipient SBW25-Tn*6291*::KmR. Three separate mixes were established for each donor-recipient combination. Cultures were incubated overnight before samples were diluted and spread onto KB agar supplemented with mercury and kanamycin. Plates were assessed after 48 h of growth at 28 °C. Single isolated transconjugants were re-streaked onto selective media, and isolated colonies were used to inoculate KB broth, which was supplemented with glycerol (20% w/v) after overnight growth, and frozen at −80 °C. Putative transconjugants were screened by PCR using plasmid-specific and recipient-specific primer pairs (Table S5, available in the online Supplementary Material). PCR was conducted with GoTaq Green G2 mastermix (Promega: M7822) using a 95 °C denaturing temperature and annealing temperature of 58 °C, both for 30 s, and an extending temperature of 72 °C for 1.5 min, with these steps repeating 30 times. A similar protocol was used to generate *P. fluorescens* SBW25-Tn*6291*::KmR(pP19E3.1). These strains gave an initial indication as to plasmid mobility and acted as donors in the subsequent assay to calculate transfer rates.

To measure plasmid conjugation and transposon transfer rates, single colonies of SBW25-Tn*6291*::KmR plasmid donor strains and *P. fluorescens* SBW25::SmR-*lacZ* recipient strains were separately cultured in 5 ml KB broth and incubated overnight. Each donor was mixed with a recipient at varying volumetric ratios to achieve approximate 1 : 1 start count ratios of donor and recipient. Three separate mixed cultures were made for each donor-recipient combination to produce three independent replicates, with two independent transconjugants tested for each plasmid. KB broth (5 ml) was then inoculated with 50 µl of the mixture. Start counts of donor and recipient were obtained by diluting and spreading samples of mixed culture onto KB agar containing X-gal. The *lacZ* gene enabled recipients to be distinguished from donors by the production of a blue pigment on X-gal-containing media. Cultures and plates were incubated overnight, after which the cultures were diluted and spread on selective and non-selective KB plates, also containing X-gal, to enumerate donors and recipients (non-selective plates), plasmid transconjugants (streptomycin and mercury supplementation) and transconjugants that had also received Tn*6291*::KmR (streptomycin and kanamycin supplementation). Single colonies of putative transconjugants, from the selective plates for both plasmid transfer and transposon transfer, were re-streaked on selective media, and PCR (as previously described) was used to ascertain the presence of the Tn*6291*::KmR and plasmid.

To transfer the counts into rates, the following equation was used [75]:


$$\gamma =\varphi \ln\left(1+\frac{T}{R}.\frac{N}{D}\right).\frac{1}{\left[N-N_{0}\right]}$$

γ refers to the transfer rate (ml cell^−1^ h^−1^) (for either plasmid conjugation or transposon transfer), Ψ is the growth rate in (h^−1^); T, R and D are the number of transconjugants (or transposon transfers), recipients and donors, respectively, per ml. N is the final number of colony forming units per ml (c.f.u./ml) in the population, and N_0_ is the starting number of c.f.u./ml in the population. The Simonsen equation assumes that donor, recipient and transconjugant growth rates are equal, which is unlikely to be the case owing to known fitness costs of the pQBR plasmids [40]. However, the approximate extended Simonsen method (ASM) [76], a more precise method, previously produced similar conjugation rates under our experimental conditions [38], and pilot studies indicated that our ASM protocol lacked the sensitivity to reliably detect transposon transfer events.

### Measuring plasmid fitness effects

Competitive fitness experiments were performed as described by Hall *et al*. [40], with details provided in Supplementary Methods, alongside details of growth curve analyses.

### Statistics

Data analysis was conducted in R [54] using the following packages: Tidyverse [55], forcats [77], multcomp [78] and FSA [79]. Figures were plotted using ggplot2 [80], patchwork [81] and ggtext [82].

The ratio of transposon transfer compared to plasmid transfer was calculated by dividing γ for transposon transfer by γ for plasmid transfer and log-transformed. If transposon transfer rates were lower than the limit of detection, their value was replaced by the limit threshold value before log-transformation. The effect on the ratio by plasmid and plasmid group and the effect of conjugation rate on transposon transfer rate were investigated using Spearman’s correlation and linear models with Tukey HSD post-hoc testing.

Comparative fitness values, w, were corrected by dividing by the mean of the control fitness values. Control values were not significantly different from 1 (mean w=1.018; T-test, t=0.84, df=9, *P*=0.42). Corrected comparative fitness values were analysed using ANOVA with Tukey HSD post-hoc testing and plotted.

## Results

### Sequence analysis of pQBR plasmids reveals four groups of large plasmids capable of frequent, active transfer of TEs

**Fig. 1.**
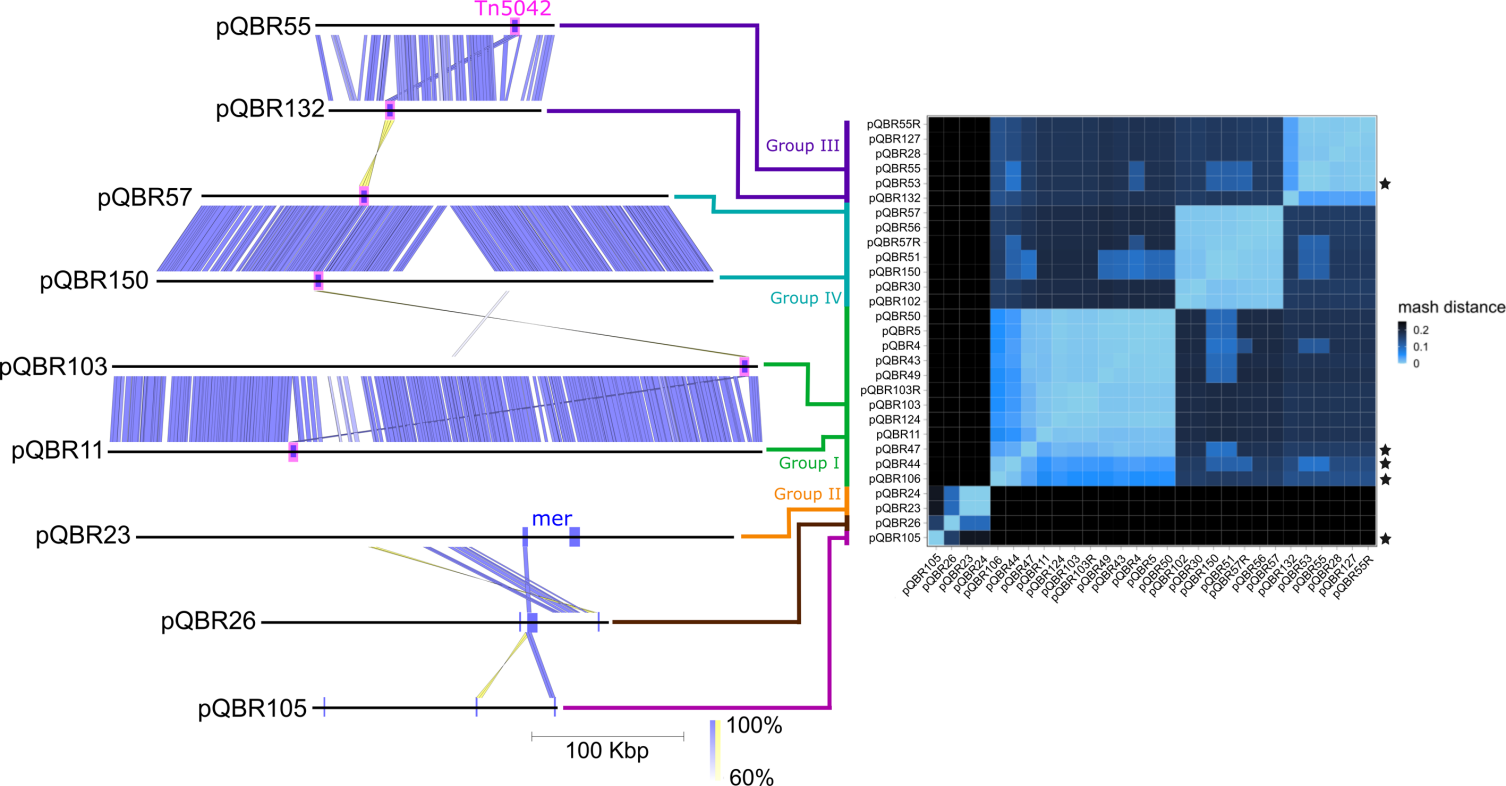
Sequence-based comparison of the pQBR plasmid collection shows distinct plasmid groups. On the right, a Mash distance heatmap of all resolved plasmid sequences, with the brightness of each tile corresponding to the genetic similarity between each pair of plasmids. Stars indicate plasmids we were unable to conjugate into *P. fluorescens* SBW25 under our experimental conditions. On the left, a diagram showing representatives of each group indicates a high degree of conservation within each group and a high degree of divergence between each group. Polygons indicate tblastx hits between adjacent sequences, with the intensity of the blue or yellow indicating sequence identity in the forward and reverse directions, respectively (tblastx hits filtered for >60% minimum identity, E<1e-1000, 500 bp length of blast match). The location of the predominant mercury resistance transposon, Tn*5042*, is highlighted in pink, and *mer* genes are indicated in blue.

The pQBR collection is a diverse group of mercury resistance plasmids captured from the same site in Oxfordshire by exogenous isolation over 3 years, using *P. putida* UWC1 and *P. fluorescens* SBW25 recipients [25,27]. Using whole-genome sequencing on 28 strains, including 24 with previously unsequenced plasmids, we generated 20 complete plasmid assemblies and 5 draft assemblies. These were supplemented with the previously sequenced pQBR44 to give a total of 26 sequences for analysis (Table S1). The pQBR plasmids were originally classified into five groups (I–V) based on restriction fragment length polymorphism [25]. At a global level (Fig. 1), k-mer-based comparison of the resolved plasmid sequences revealed that most of the new plasmid sequences (19 out of 22) fell into these previously assigned groups (Table S1). With few exceptions, plasmids within each group had a high degree of identity and sequence similarity, whereas between groups, there was little overall sequence similarity (Fig. 1).

Plasmid pQBR103 and, by extension, the Group I pQBR plasmids, were recently proposed to form a distinct group of *Pseudomonas* plasmids, termed PInc-17 or pQBR103-like [83]. To investigate whether any of the other pQBR groups corresponded to the PIncs described by Nishimura *et al*. [83], we compared sequences to the canonical sequences of each of the PIncs. The Group II plasmids produced significant matches to the PInc-18/Inc_PSTY_ group (56% blastn coverage at >80% identity). There were no substantial PInc matches for Group III (pQBR55-like) plasmids or Group IV (pQBR57-like) plasmids, which therefore represent a novel grouping in the *Pseudomonas* plasmid PInc taxonomy. Plasmid pQBR26, which had previously been designated Group II but is considered on the basis of sequence evidence presented here to represent a distinct pQBR group, matched the PInc-7/IncP-7 group (42% coverage).

**Fig. 2.**
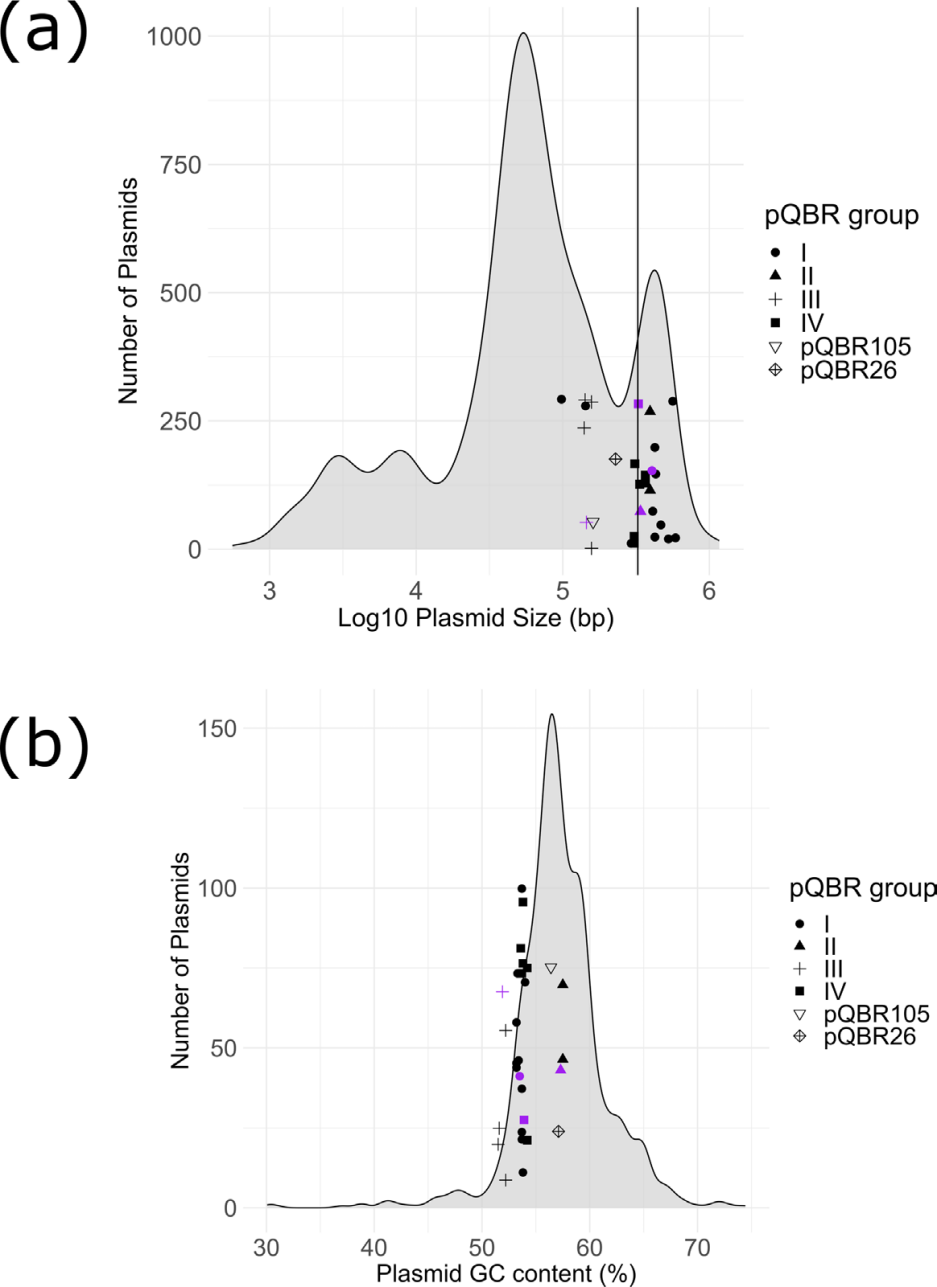
(a) Plasmids within the pQBR collection are generally large. The size of each resolved pQBR plasmid is compared with a density plot of *Pseudomonas* plasmids in PLSDB (v.2024_05_31_v2). Vertical line represents 5% of median genome length of the complete genomes on PseudomonasDB. (**b) **GC content (%) of pQBR plasmids compared to *Pseudomonas* plasmids within PLSDB. In both figures, plasmids from pQBR collection are represented by points jittered on the y-axis for visibility, and group averages are indicated by purple points.

The sequenced pQBR plasmids were generally large, and most are on the boundary of being megaplasmids [84], ranging in size from 140 kb (176 CDS, pQBR132) to 588 kb (712 CDS, pQBR49), and constituting 2.1–9.0% of the median genome size for *Pseudomonas* species [85], respectively (Fig. 2a). All possessed a GC content lower than the average for *Pseudomonas* genomes (Wilcoxon test, *P*<2.2e-16 for all plasmids; Fig. 2b). Furthermore, the pQBR plasmids are evidently mobile: most displayed conjugation between strains in laboratory settings in addition to the initial mobilization into the exogenous isolation recipient (Table S1); thus, they should not be considered non-transmissible ‘chromids’ or sedentary secondary chromosomes.

At the nucleotide level, few genes were conserved across plasmids from different groups. The remarkable exception was the presence of highly similar TEs found on plasmids from different groups. Most prominent was Tn*5042*, a 6,989 bp transposon with a *merRTPCAB* operon, previously identified on pQBR103, pQBR57 and pQBR55, and here found on all the other plasmids except Group II, pQBR26 and pQBR105. These copies of Tn*5042* all possessed high sequence similarity and similar nucleotide variants compared to the reference sequence (AJ563380.2) with few exceptions (see Supplementary Results). This strongly suggests that the transposon has moved relatively recently through the local plasmid population [25]. In Group II, the mercury resistance transposon Tn*5046* (10,118 bp) was identified, sharing *merRTPCA* with ~80% nucleotide similarity to Tn*5042*, and encoding *merDE* instead of *merB*. No intact mercury resistance transposon with matches in TnCentral could be identified within pQBR26 or pQBR105, although unannotated mercury transposons are likely present. The three pQBR plasmids that were lost before sequencing owing to chromosomal *mer* capture (pQBR1, pQBR8 and pQBR58, described in Supplementary Results) each left a transposon resembling the known *Pseudomonas* mercury resistance transposon Tn*512* [86] with >97% identity and >98% coverage, suggesting that this transposon might be more prone to chromosomal capture than those discussed above that remained plasmid-borne.

Three other full-length TEs were identified on plasmids across different groups: Tn*4652* (GenBank accession AF151431.1, 17,029 bp), Tn*6290* (BK010246.1, 42,031 bp) and Tn*6291* (BK010245.1, 22,336 bp). These were generally well-conserved across the pQBR plasmids, although some contained minor divergences from their respective reference sequences (Table S2). These transposons are large, each with a cargo of genes with functions associated with metabolism, metal resistance and/or transportation (see Supplementary Results). Transposons were sometimes present in multiple copies; most strikingly, Tn*6290* was present in three copies in pQBR49. It is likely that these transposons were acquired during exogenous isolation as Tn*4652* and Tn*6290* are present in the *P. putida* UWC1 chromosome, whilst Tn*6291* is present in *P. fluorescens* SBW25 [25,29] (details on putative mechanisms of transposition are provided in Supplementary Results). Of the 26 pQBR plasmids we analysed in the collection, 11 harboured isolation-associated TEs, demonstrating the readiness with which these MGEs can transfer chromosomal elements and indicating the ability of pQBR plasmids to act as vehicles for horizontal gene transfer.

Using AMRFinderPlus, we did not identify any AMR genes within the pQBR collection and found no increased resistance conferred by any of the pQBR plasmids against colistin, kanamycin, streptomycin or tetracycline in *P. putida* UWC1. The pQBR plasmids thus constitute a set of experimentally tractable, ecologically cohesive plasmids originating from a non-AMR, non-clinical context.

### Group I and IV pQBR plasmids have gene conservation despite extensive divergence and are distant relatives of AMR plasmids

**Fig. 3.**
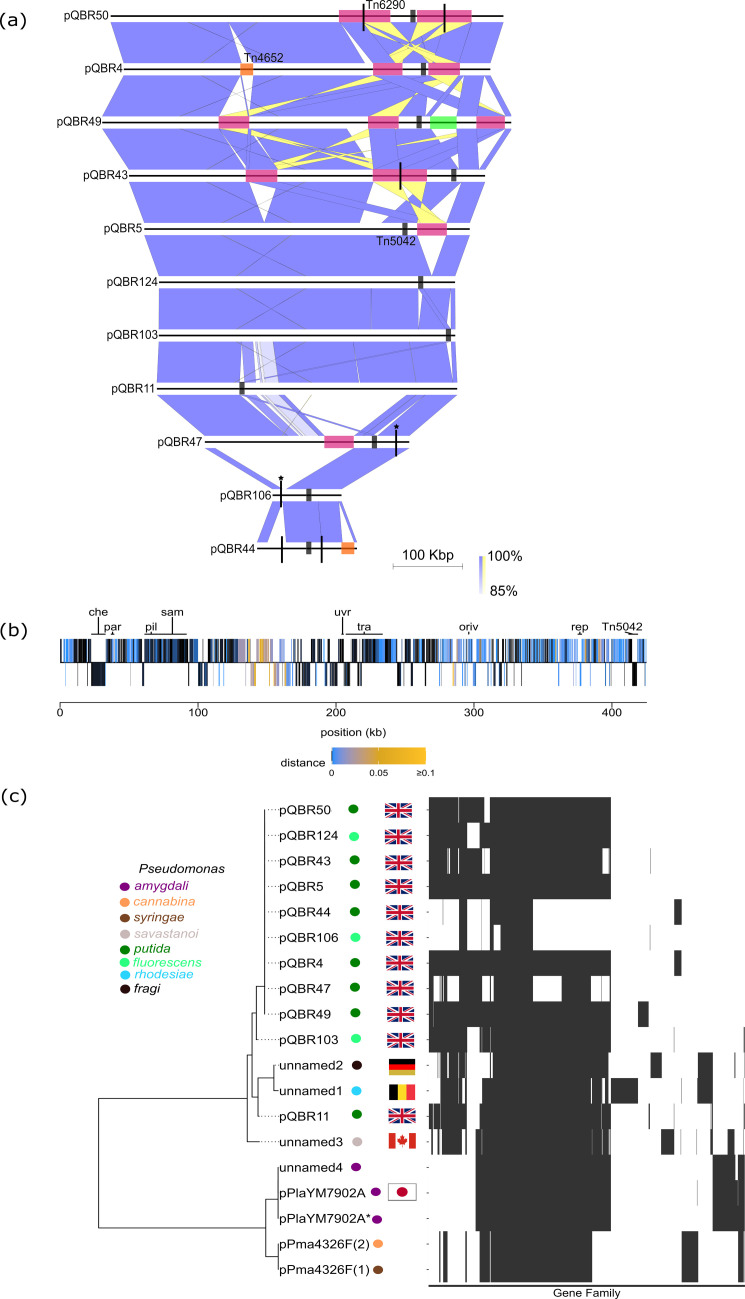
Group I pQBR plasmids share a conserved backbone but have experienced high levels of transposon activity alongside truncations. (**a)** blastn comparison of Group I pQBR plasmids, with transposons highlighted in pink (Tn*6290*), orange (Tn*4652*) and black (Tn*5042*). The green highlighted region in pQBR49 is the unique region containing Erebus defence system. Lines extending above and below lines on these sequences indicate location of contig breaks, due to Tn*6290* presence interfering with assembly, and lines with a star above indicate breaks due to potential chromosomal integration. (**b)** Comparison of gene conservation amongst Group I plasmids. Raw nucleotide divergence (p-distance) was calculated across homologues for each pQBR103 gene. Genes with higher divergence across the group are shown in more intense shades of yellow, less divergence in blue and invariant genes coloured in black. Genes above or below the line represent genes on the forward or reverse strand. (**c)** Phylogenetic tree of the core genome and heatmaps showing gene presence/absence across the Group I plasmids and relatives from PLSDB and PseudomonasDB. Note that pPma4326F (1) and pPma4326F (2) are from different host strains, *P. syringae* pv*. maculicola* (NZ_CP047261.1) and *P. cannabina* pv*. alisalensis* (NZ_CP084324.1), respectively.

The Group I plasmids (*n*=11, including the draft assemblies) were on average the largest plasmids in the pQBR collection, ranging between 98 and 588 kb, with a mean of 406 kb (Table S1). As previously observed for pQBR44 [40], plasmids pQBR47 and pQBR106 are likely to represent truncated variants: these three plasmids were substantially smaller than the others in the group, lack several of the key functional regions predicted for other Group I plasmids (including conjugative transfer) and were not self-mobilizable in our experimental assays (Table S1). The remaining Group I plasmids shared extensive identity and synteny, with most structural variation associated with TEs (Fig. 3a). Mercury transposon Tn*5042* was located in a syntenic region in all Group I plasmids, except for pQBR103 and pQBR11 in which the locations were unique. Plasmids pQBR4, pQBR5, pQBR43, pQBR47, pQBR49 and pQBR50 carried transposon Tn*6290*, sometimes with multiple copies. Most of these were in different locations, but pQBR4, pQBR47, pQBR49 and pQBR50 had at least one conserved insertion site (Fig. 3a).

Examining neighbouring genes to each Tn*5042* copy revealed that TE acquisition likely disrupted plasmid genes: an SmpA-OmlA domain-containing protein, which was intact in pQBR11 (PQBR11_02450) and pQBR103 (PQBR103_02355), was present only in truncated or pseudogenized form due to the insertion site shared by pQBR4 and relatives. TE insertion appeared to have disrupted a Transglut-core3 domain-containing protein in pQBR11 (PQBR11_00620), and in pQBR103, a DUF913 domain-containing protein (PQBR103_02645). An additional orphan ParB protein was noted in pQBR103 and pQBR11, and pQBR49 contained a unique region, encoding putative phage defence system Erebus (Fig. 3a) [87]. The region unique to pQBR49 also contains several phage/MGE-related genes, including a holin (PQBR49_03095), a phage protein (PQBR49_03025), four Tyr recombinases (PQBR49_03030, PQBR49_03045, PQBR49_03055 and PQBR49_03060) and a transposase (PQBR49_02960), suggesting that this region is a degraded remnant of another MGE (no intact phages were detected on any plasmids). MOBsuite could not identify the mobility or relaxase genes within Group I or match them to known MOB gene families, but most Group I plasmids were experimentally conjugated into *P. fluorescens* SBW25 successfully (Table S1).

Using the backbone sequence of pQBR103 as a query, we used Mash [57] to search public databases for related plasmids. We found 13 complete plasmid sequences with an average of 85–93% nucleotide identity to pQBR103, with a 300–400 kb shared backbone in non-truncated variants. All sequences were associated with *Pseudomonas* spp., and most originated from plant-associated environments or contaminated soils, except for two: a *P. rhodesiae* plasmid and a *Pseudomonas fragi* plasmid isolated from a brewery [88] and raw milk, respectively [89] (Table S3).

The Group IV plasmids (*n*=6) ranged between 307 and 366 kb (328 kb average) and were all conjugative (Table S1). The six plasmids showed extensive identity and synteny, alongside TE activity. Tn*5042* was present in syntenic positions in pQBR51, pQBR56, pQBR57 and pQBR150, potentially disrupting an efflux RND transporter periplasmic adaptor subunit and flanked by DNA helicase and a transcriptional regulator. A Tn*5042* insertion site was shared between pQBR30 and pQBR102, with no obvious gene disruption when compared with a syntenic site on the other plasmids. Two plasmids (pQBR51, pQBR150) contained Tn*4652* and Tn*6290* in the same location and tandem arrangement, whereby Tn*4652* appeared to have inserted within Tn*6290* at base 40,829 in the transposon reference sequence (BK010246.1) to generate a nested structure. Tn*6291* was found only in pQBR102, flanked by a DNA helicase. Beyond TE activity, minor variation in gene content mainly came from pQBR30 and pQBR102 (details in Supplementary Results).

As with the Group I plasmids, we attempted to identify related plasmids for Group IV and found only a draft *P. putida* genome of unknown origin (NZ_JANHLM010000005.1). As with Group I, MOBsuite was unable to predict mobility correctly for Group IV, and relaxases could not be matched to known MOB gene families.

The Group I and Group IV plasmids each harbour a conserved backbone ~200 kb in length. We investigated sequence variation across the conserved genes (Figs3b  4b). The most divergent genes across Group I were mainly genes of unknown function, several of which encoded putatively membrane-associated proteins. Within Group IV, divergent genes included a transcriptional regulator, a serine/threonine protein kinase, a putative bacteriophage protein, a YrdC-like domain-containing protein and two leader peptidases. These may represent more rapidly evolving loci, perhaps involved in coevolutionary interactions, but the unknown function of these genes makes further speculation difficult.

**Fig. 4.**
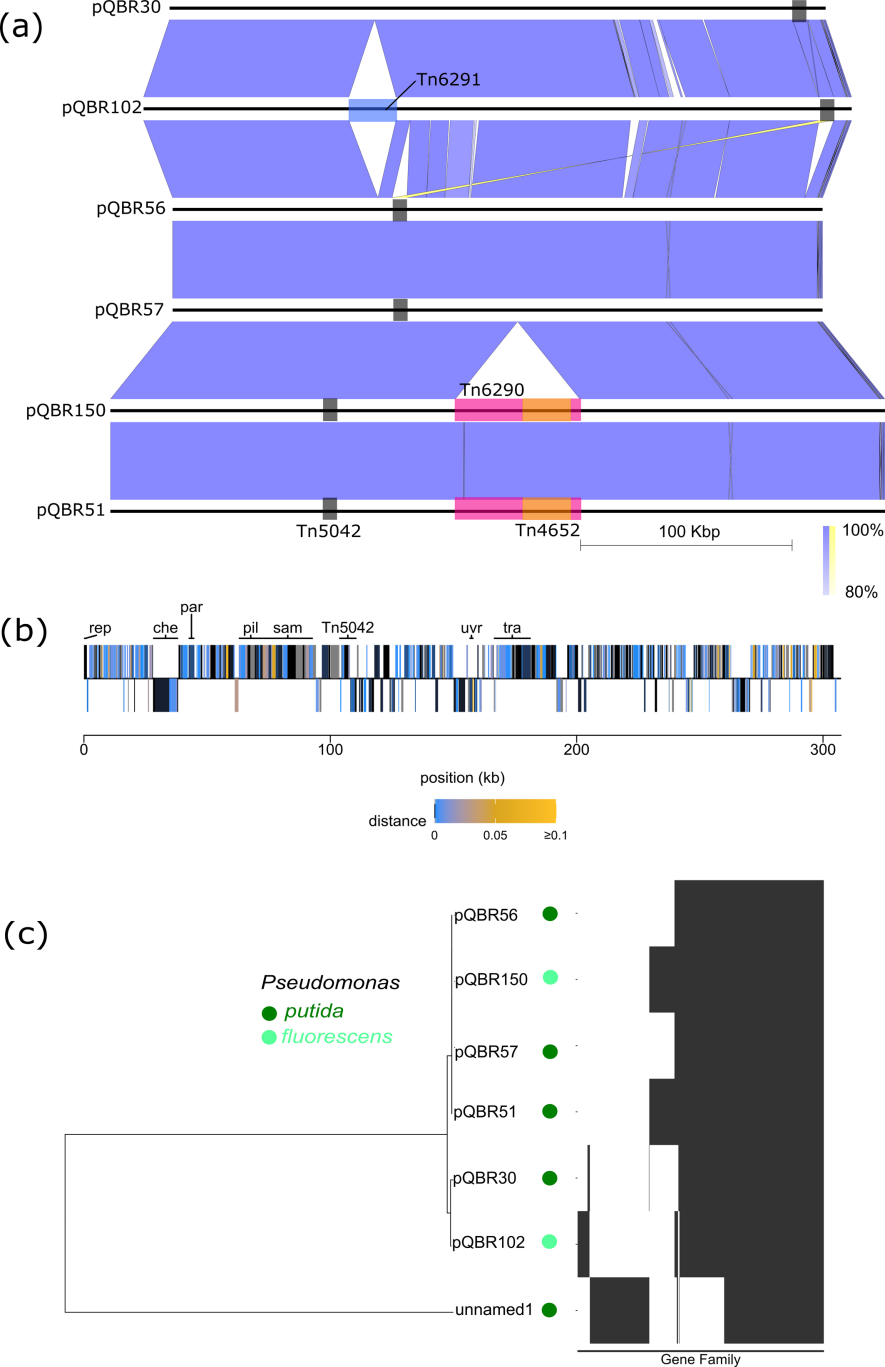
Group IV pQBR plasmids shared a conserved backbone. (**a)** blastn comparison of Group IV pQBR plasmids, with transposons highlighted in blue (Tn*6291*), pink (Tn*6290*), orange (Tn*4652*) and black (Tn*5042*). (**b)** Comparison of gene conservation amongst Group IV plasmids. Raw nucleotide divergence (p-distance) was calculated across homologues for each pQBR57 gene. Genes with higher divergence across the group are shown in more intense shades of yellow, less divergence in blue and invariant genes coloured in black. (**c)** Phylogenetic tree of the core genome and heatmaps showing gene presence/absence across the Group IV plasmids and the relative identified in PseudomonasDB.

**Fig. 5.**
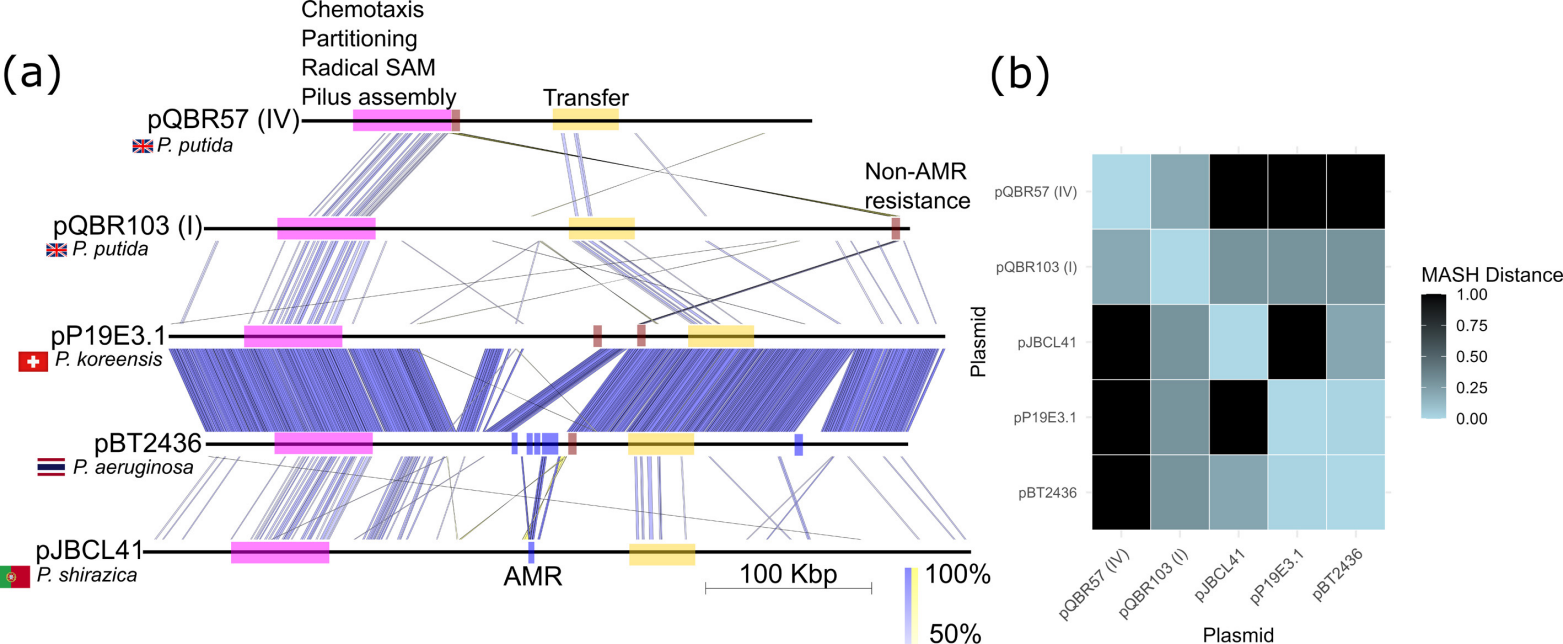
Group I and Group IV plasmids have divergent, globally dispersed relatives. A comparison of plasmids pQBR103 (Group I), pQBR57 (Group IV), p1 from *P. koreensis* P19E3 and pBT2436 from *Pseudomonas aeruginosa* BT2436 (IncP-2) and pJBCL41 from *P. shirazica* FFUP_PS_41. (**a)** tblastx comparison of plasmids (>50% minimum identity, E<1e-1000, minimum blast match length 200 bp) with shared regions of interest highlighted. Antibiotic resistance (AMR) and non-AMR resistance genes, as predicted by AMRFinderPlus, are highlighted in dark blue and red, respectively. (**b)** Heatmap of sequence similarity based on Mash distance.

Though divergent at the nucleotide level, the Group I and Group IV backbones contain many similar genes arranged in a similar order, encoding putative functional pathways such as a chemotaxis phosphorelay system, a type IV pilus and radical SAM-metabolizing enzymatic clusters [40]. Divergent, yet similar, backbone regions were also found to be present in the IncP-2 plasmids, including the multi-drug resistance plasmid pBT2436 and the environmental megaplasmid p1 (from strain *P. koreensis* P19E3) [11], and the *P. shirazica* plasmid pJBCL41 [90]. We compared the backbone regions of Group I, Group IV, IncP-2 and pJBCL41-like plasmids, and whilst highly divergent at the nucleotide level (43–50% nucleotide identity), tblastx comparisons clearly highlighted clusters of 53 and 33 kb of homologous genes with similar predicted functions (Fig. 5). Together, our results show an ancient plasmid backbone shared amongst environmental and clinical plasmids worldwide, capable of readily transferring traits, including antibiotic and metal resistance, between hosts.

### Sequence comparisons of other pQBR plasmid groups

**Fig. 6.**
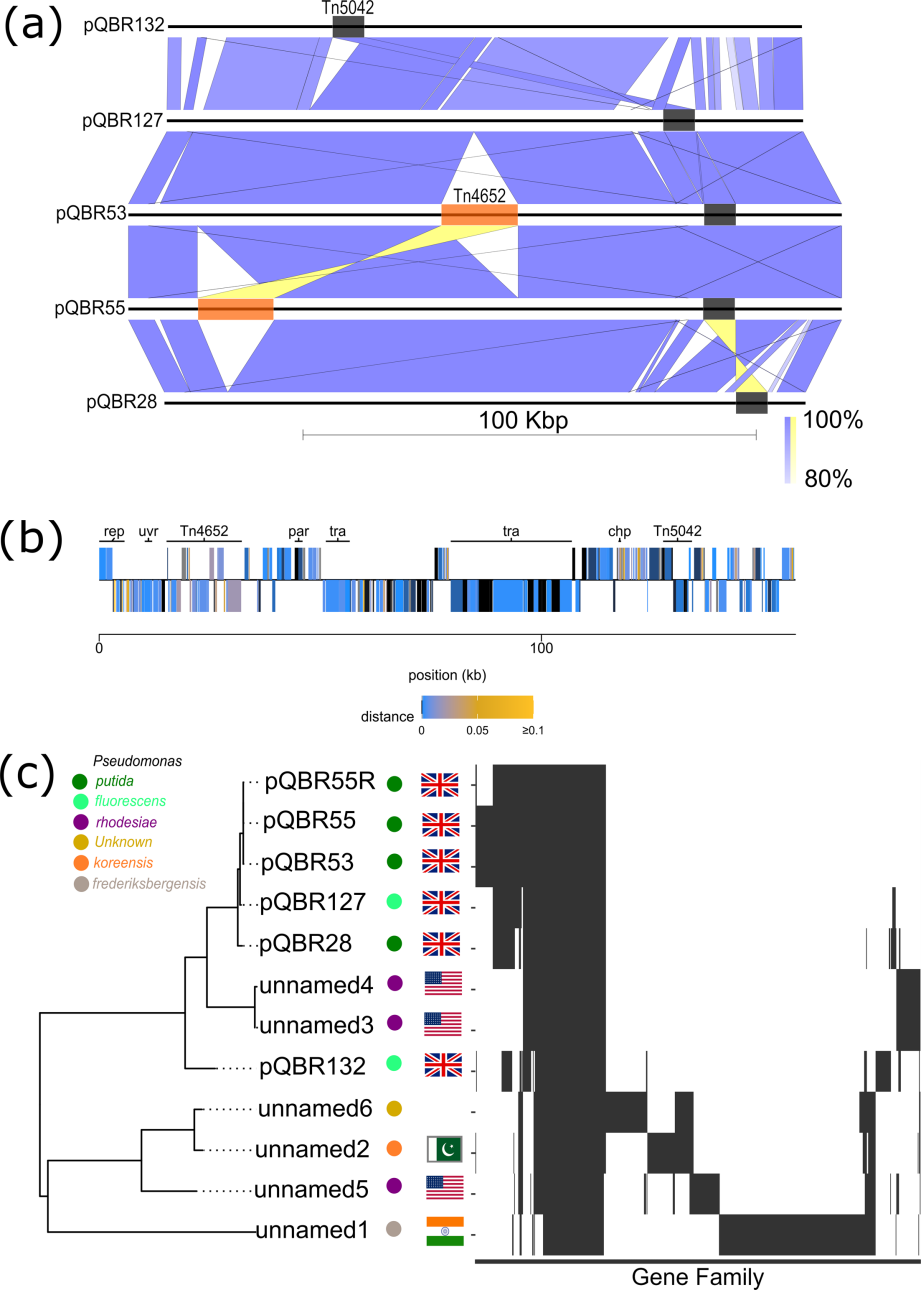
Group III pQBR plasmids show more backbone divergence than Groups I and IV. (**a)** blastn comparison of Group III pQBR plasmids, with transposons highlighted in orange (Tn*4652*) and black (Tn*5042*). (**b)** Raw nucleotide divergence (p-distance) was calculated across homologues for each pQBR55 gene. Genes with higher divergence across the group are shown in more intense shades of yellow, less divergence in blue and invariant genes coloured in black. (**c)** Phylogenetic tree of the core genome and heatmaps showing gene presence/absence across the Group III plasmids and relatives from PLSDB and PseudomonasDB.

In Group III (*n*=4), plasmids ranged from 140 to 157 kb (mean 145 kb). Again, we identified TE activity (from Tn*5042* and Tn*4652*), and, in contrast to Groups I and IV, significant divergence in the backbone sequences, particularly with pQBR132, which had lost various genes from across the plasmid and showed lower overall sequence identity (Fig. 6a). Of note, pQBR132 did not encode a MazEF toxin–antitoxin system shared amongst other Group III plasmids (Table S1), whilst the genes for paREP8 (potentially involved in cell defence [91]) and a dystrophin (pQBR132_00470) were apparently truncated. Tn*5042* was present in a syntenic location within pQBR53, pQBR55 and pQBR127, except for a DNA-binding protein in pQBR127 which may have been disrupted by the transposon (PQBR127_00665). The other two Group III plasmids have different insertion site locations. Plasmid pQBR53 in Group III has transposon Tn*4652* inserted into a putative *traC* CDS and pilus assembly protein which may have impacted its ability to conjugate, as it was the only Group III plasmid we were unable to conjugate into *P. fluorescens* SBW25 (Table S1). The most divergent genes across the Group III plasmids were related to DNA processing, transcriptional regulation and metabolism (Fig. 6b).

We identified six unnamed plasmids in public databases that matched Group III, mainly matching with putative replication and transfer loci. Using tblastx, these related plasmids shared core function genes, including partitioning, conjugal transfer, regulatory and DNA replication genes. The two plasmids of known origin, from *P. koreensis* and *P. frederiksbergensis*, were isolated from soil and a glacial stream, respectively (Table S4). *P. resinovorans* plasmid pCAR1 (AB088420), previously identified to share some matching regions with pQBR55 [40], had greater similarity to pQBR26, with all three encoding MOBH relaxases.

**Fig. 7.**
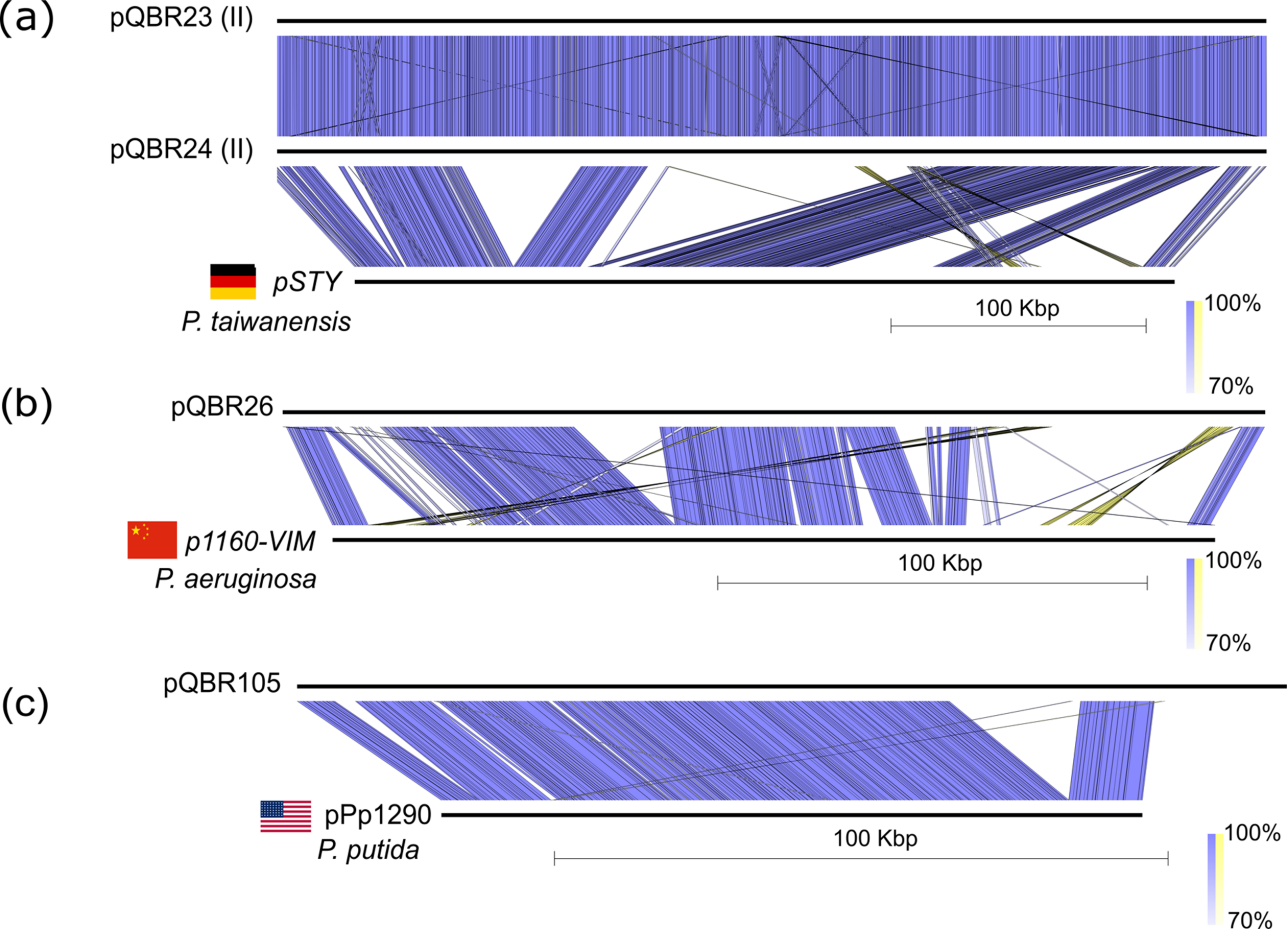
Group II, pQBR26 and pQBR105-like plasmids have globally dispersed relatives identified by mob_typer.** (a)** tblastx comparison of Group II plasmids pQBR23 and pQBR24 with *P. taiwanensis* VLB120 pSTY. (**b)** Plasmid pQBR26 and *P. aeruginosa* p1160-VIM. (**c)** Plasmid pQBR105 and *P. putida* pPp1290. All tblastx comparisons were filtered >70% minimum identity, E<1e-1000, minimum blast match length 100 bp.

The Group II (*n*=2) plasmids [pQBR23 and pQBR24, average nucleotide identity [92] (ANI>99.9%)] were highly similar and contained multiple toxin–antitoxin systems and defence systems (Table S1). Both plasmids were predicted to be conjugative by MOBsuite, encoding MOBF and MOBP relaxases. MOBsuite also predicted that Group II was related to *Pseudomonas taiwanensis* VLB120 pSTY (isolated from forest soil, Germany) (Fig. 7a). Plasmid pQBR26 was previously placed in Group II [25], but the resolved pQBR26 sequence showed similarity to the other Group II plasmids only in accessory genes and putative TEs. We therefore assigned it to a novel grouping. MOBsuite predicted that pQBR26 was related to carbapenemase-encoding *P. aeruginosa* plasmid p1160-VIM (China) (Fig. 7b) and that it was conjugative with a MOBH relaxase. Sequence analysis identified multiple toxin–antitoxin and defence systems (Table S1), including a CRISPR-Cas system (subtype IV-A) and an anti-CRISPR system (Table S1). Spacer sequences from the CRISPR-Cas in pQBR26 revealed matches to genomes of plasmids in *P. aeruginosa* and *P. paraeruginosa* (CP173144.1, CP029091.1 and CP027167.1, 128–166 kb in size) and one *P. rhodesiae* chromosome (LT629801.1). Comparison of these plasmids to representatives from the pQBR groups (pQBR57, pQBR103, pQBR55, pQBR23, pQBR26 and pQBR105) showed minimal nucleotide similarity, suggesting that the pQBR26-encoded CRISPR-Cas is involved in targeting dissimilar plasmid competitors.

Plasmid pQBR105 did not share enough relatedness to any of the other pQBR plasmids to be classified into Groups I–IV. Although containing mercury resistance genes and many transposases, we were unable to identify any known transposons registered with TnCentral. Plasmid pQBR105 is predicted to be conjugative by MOBsuite, with MOBP relaxases identified and relatedness to *P. putida* pPp1290 isolated from an orchard leaf in California, USA (Fig. 7c), but we were not able to mobilize it to *P. fluorescens* SBW25 in our assays.

### Plasmid transfer of chromosomal traits varies across pQBR plasmids and is not determined by conjugation rate

Previously, pQBR57 was observed to be an effective vehicle for inter-species chromosomal transposon transfer [20]. The presence of diverse TEs across different pQBR plasmid groups indicates that these plasmids may be effective vehicles for acquiring and disseminating TE-borne traits. To investigate the capacity of different pQBR plasmids to transfer a chromosomally encoded TE to a new host, we tracked the transfer of Tn*6291* from the *P. fluorescens* SBW25 chromosome to a naive host. Using differential selective markers, we were able to distinguish transconjugants carrying the focal plasmid, and those carrying the focal plasmid which had also acquired the transposon. Alongside the pQBR plasmids, we included the plasmid pP19E3.1 [45], which shares distant backbone similarity to pQBR57 and pQBR103 and has close relatives implicated in the global spread of antibiotic resistance, as described above.

**Fig. 8.**
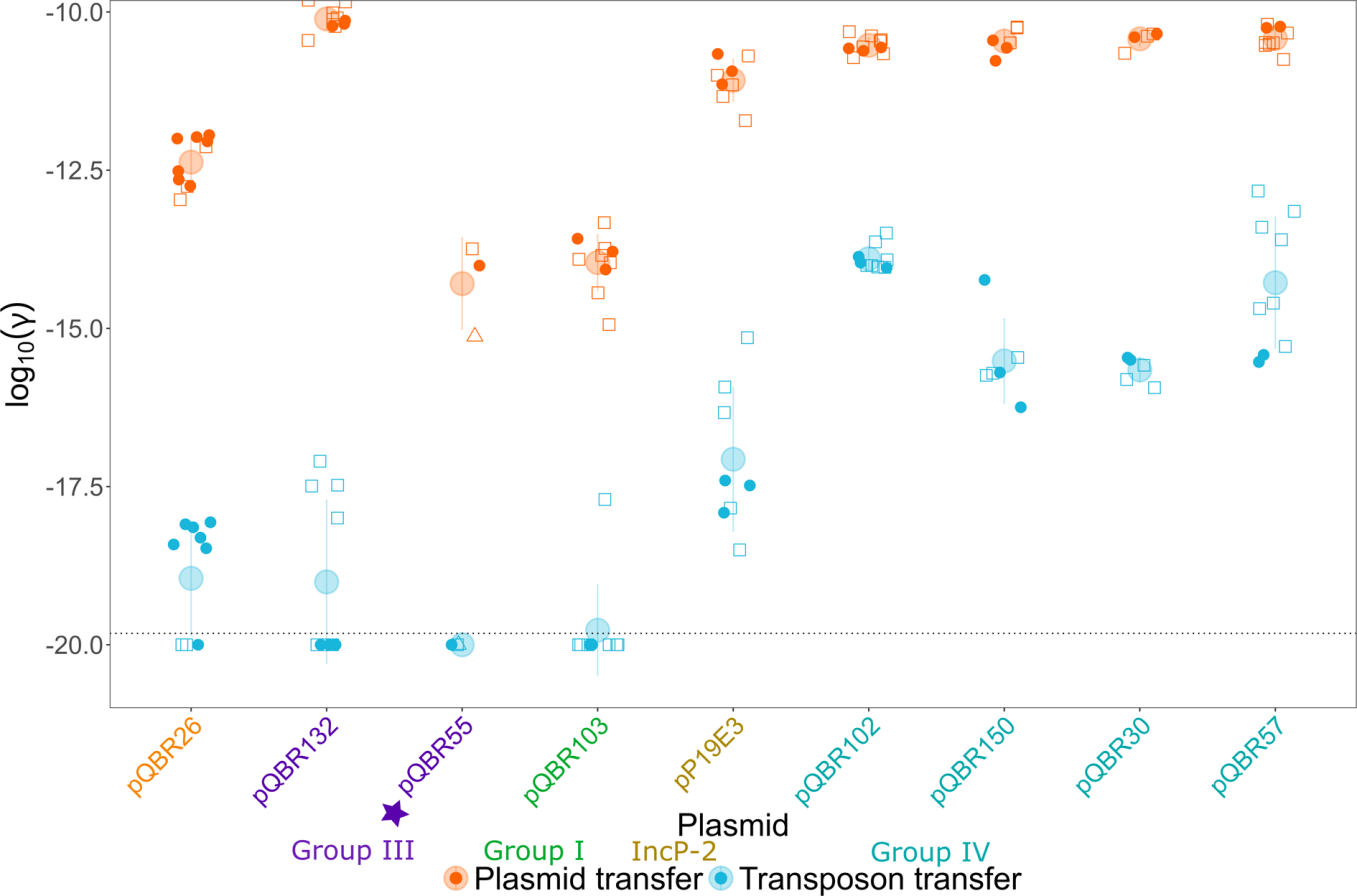
Conjugation rate is not a good predictor of transposon transfer. The plot shows transposon transfer rates (blue) and plasmid transfer rate (orange). Dotted line represents the estimated limit of detection, calculated using the average growth rate and if a single transconjugant colony was counted. Large circular datapoints represent the mean of log10-transformed transfer rate values for that plasmid, and bars represent the standard deviation. This figure represents a summary of two experiments performed a month apart, with two to three different donor transconjugant strains for each plasmid. Colours of plasmid labels represent the group the plasmid belongs to, labelled beneath. Star (pQBR55) indicates that data for this plasmid were collected separately from the rest of the dataset under similar experimental conditions.

We hypothesized that transposon transfer would vary with conjugation rate, with the most conjugative plasmids being most effective at transferring Tn*6291*. However, though we detected an overall correlation (Fig. 8, Spearman’s correlation rho=0.5, *P*<0.001), this was driven by the two plasmids with the lowest conjugation rates that had similarly low transposon transfer rates (pQBR103, pQBR55). Once these plasmids were removed, there was no significant relationship between conjugation rate and transposon transfer rate (rho=0.25, *P*=0.07), with ratios of plasmid conjugation rate to transposon transfer rate varying between plasmids and plasmid groups by several orders of magnitude [Fig. S1, F_(8,62)_=34.34, *P*<0.01]. For example, pQBR132 (Group III) had a strikingly lower ratio compared with the other plasmids (*P*<0.01), being ineffective at transferring Tn*6291* despite a relatively high conjugation rate, whilst pQBR102 and pQBR57 (both Group IV) were generally most effective at mobilizing Tn*6291* with a Tn*6291*::KmR acquisition every ~2,359 pQBR102 transconjugants or ~7,255 pQBR57 transconjugants. To investigate whether transfer rate correlated with plasmid costs, we performed growth curves and competitive fitness assays but did not identify any correlation between plasmid fitness effects and the efficiency of the plasmid in transferring a chromosomal locus, suggesting that the striking differences in transposon acquisition and transfer are due to more specific differences in plasmid activity or gene content (Fig. S2).

## Discussion

The pQBR plasmids were isolated from a field growing sugar beets in Oxfordshire, UK [25,26, 31], in the 1990s and represent a collection of environmental, co-occurring plasmids that do not encode antibiotic resistance genes. This collection therefore represents a valuable system for understanding the natural ecology of ‘wild’ plasmids from non-clinical settings. Here, we have analysed 26 pQBR plasmids, including 22 novel sequences, providing a detailed insight into plasmid genetics alongside conjugation and gene transfer phenotypes, illuminating the diversity that can be found both within a plasmid community and between closely related plasmids, as well as the rapid plasmid evolution that can occur through the activity of TEs.

The pQBR plasmids are relatively large, consistent with other plasmids from soil [93]. Genome-based clustering satisfyingly recapitulated prior RFLP-based analyses [25] to place the sequenced plasmids into distinct groups. Superficially, these analyses suggested that pQBR plasmids of different groups have little in common beyond their ability to confer mercury resistance. However, closer analyses found distant resemblances when comparing plasmids of Groups I and IV despite low nucleotide identities, and similarly, both of these groups had distant similarities with both the clinically important PInc-2/IncP-2 resistance plasmids and the AMR megaplasmid pJBCL41. The presence of similar genes in a similar order — but highly diverged at the nucleotide level – speaks to ancient conservation of function across these distant plasmid taxonomic units, which are found in environments, including the infected lung [11,90], industrial processing [89,94] and soil habitats, and across diverse species of *Pseudomonas* (Figs3  5 and Table S3). Interestingly, these ancient conserved genes seem to encode features that are not obviously directly related to plasmid fitness, including chemotaxis, adherence and motility (type IV pilus) and arylsulfatase/radical SAM/iron-sulphur cluster metabolism. Whilst the functional role of these genes is yet to be determined experimentally, this observation highlights that plasmids likely have long-standing and nuanced life histories that extend beyond an existence as either simple vectors of functional traits or entirely selfish parasitic agents.

A previous comparison of plasmids from the pre- and post-antibiotic eras showed that 77% of pre-antibiotic era plasmids continued to circulate and maintain a recognizable backbone in modern times, in many cases having acquired one or more resistance genes [24]. We did not identify any known AMR genes on any of the pQBR plasmids, and plasmid carriage did not affect any antibiotic resistance phenotypes tested. The pQBR collection therefore represents a tractable set of modern environmental plasmids that have not yet been recruited as vehicles for antibiotic resistance, opening the opportunity to explore the biology of pre-resistance plasmids and experimentally study the dynamics of AMR gene acquisition by plasmids.

Within each group, significant structural differences emerged from the variable presence of TEs. Plasmids were selected using mercury, and it is likely that, thanks to TE activity, mercury resistance spread through this local plasmid community, effectively labelling a cross-section for retrieval using exogenous isolation and providing another example of the predominant role that transposon activity can play in rapid plasmid evolution. In many cases, TEs were likely acquired during the process of exogenous isolation, as transposons identified were often present in the isolation host strains and dramatic abiotic changes associated with exogenous isolation may have triggered transposition. For example, the presence of metals within media has been shown to enhance the activity of Tn*4652* [95]. However, there was variation in the patterns of different transposons between groups, potentially hinting at barriers or limitations emerging from plasmid host range or genetic content. For example, Tn*6290* was prolific in its insertion within Group I, with 45% (6 out of 11) of the sequences containing this transposon, often with multiple copies, whereas in Group IV, Tn*6290* was found in 33% (2 out of 6) of the plasmids with a single copy, a nested variant also harbouring Tn*4652*. Whilst some molecular work has demonstrated that Tn*4652* transposes via a replicative mechanism [96], the mechanisms of Tn*6290*, Tn*6291* and Tn*5042* have not been studied directly, and differences in the regulation of the cognate transposases may have contributed to their varying proliferation within different groups (details in Supplementary Results). Overall, the presence of many TEs and transposases in these sequences, and their apparently recent and repeated acquisition, alludes to the readiness of these plasmids to acquire traits through transposition.

TE acquisition may not always be beneficial, as insertion into core regions could have deleterious effects on plasmid fitness [97,98]. Plasmid pQBR53 had Tn*4652* insert directly into genes putatively involved in horizontal transmission, and the fact that we were unable to mobilize it suggests that it became incapacitated as a consequence. Pre-existing TEs present in sequences can mitigate this risk by acting as a ‘safe’ target – a TE inserting within another is not disrupting an essential gene – and indeed, in 2 cases (Group IV), we identified nested transposons. This nesting pattern has also been observed with TEs in eukaryotes [99,100]. The activity and additional genetic material carried by TEs might also act as a burden on plasmids. Eukaryotes have defence systems that suppress the activity of TEs [101], and in bacteria, H-NS proteins can act to direct transposons away from essential genes [102]. An H-NS homologue was identified on pQBR26, which may act similarly to protect the plasmid from disruptive transposon activity.

Groups within the pQBR collection showed varying prevalence of defence systems and toxin–antitoxin systems, indicating divergent strategies for plasmid persistence. Erebus, a phage defence system with a currently unclear mechanism of action, was uniquely present on pQBR49 amid a ~37 kb region which was likely acquired by the plasmid from elsewhere, pointing to the dynamic nature of defence system reassortment on plasmids. Plasmid pQBR26 possessed a CRISPR-Cas system, with spacers matching other (non-pQBR) plasmids, whilst the Group III plasmids all possessed a vcrx091-093 anti-CRISPR system [103], consistent with an emerging role for genome defence in plasmid competition [5,104, 105]. Whilst smaller plasmids can rapidly acquire mutations to avoid restriction-modification systems, larger plasmids, like the pQBR collection, tend to acquire avoidance systems [106,107]; indeed, most of the pQBR plasmids, with the exception of pQBR106, carry orphan methyltransferases that may act to provide protection against restriction endonucleases. Defence systems can be valuable, but they can present a fitness cost and may influence the ability of a plasmid to enter new hosts and to acquire and transfer valuable new traits [108]. For example, compared with other Group III plasmids, pQBR132 lacked a region containing a predicted MazEF-like anti-phage/toxin–antitoxin system, which may explain its increased rate of conjugation compared with pQBR55 from the same group.

Different pQBR plasmids varied widely in their ability to transfer a chromosomal transposon to another host, with some related plasmids showing order-of-magnitude variation in transposon mobilization rates, despite similar conjugation rates, suggesting that another factor was in play. Previously, we found that pQBR57 and pQBR103 plasmid acquisition directly upregulated the putative transposase of Tn*6291* in a manner independent of plasmid fitness cost [35], and here, we did not find any correlation between fitness costs and transposon transfer, suggesting that unknown genetic factors can influence the rate of transposon acquisition, with some plasmids perhaps better at stimulating TEs than others, or copy numbers of TEs within the plasmid influencing expression of TE genes [109].

Our work provides a plasmid collection suitable for exploring the evolution and dynamics of gene acquisition by pre-AMR plasmids, and the diversity that can exist within and between genetically distinct plasmid groups from the same niche. This study highlights the pervasive interplay between plasmids and TEs that can drive plasmid and microbial genome evolution. Further investigations may reveal factors that promote or reduce transposition, such as TE copy number [109], potentially uncovering genetic conflicts with particular transposons, as exist with plasmids and their hosts [35]. Understanding insertion sites of transposons within these plasmids could reveal methods of plasmids mitigating deleterious insertions. Previously, it has been shown that pQBR57 (Group IV) and pQBR103 (Group I) can coexist within a host [110]. As we do not know the original hosts of these plasmids, it would be useful to establish their host range and compatibility across and within groups. A recent study has begun to explore these patterns with pQBR57, finding conjugation occurring between various *Pseudomonas* spp. and five putative defence systems that may be a barrier to pQBR57 acquisition [111]. Given the genetic diversity and variation in defence system presence across the pQBR plasmids, this collection could provide key insights into host-range dynamics and genome defence.

## Supplementary material

10.1099/mgen.0.001789 Supplementary Material 1.

10.1099/mgen.0.001789 Supplementary Material 2.

## Abbreviations

AMR: antimicrobial resistance
ASM: approximate extended Simonsen method
c.f.u./ml: colony forming units per ml
KB: King’s B media
MGEs: mobile genetic elements
ONT: Oxford Nanopore Technology
RFLP: restriction fragment length pattern
RIP: replication initiation protein
SAM: S-adenosyl-l-methionine
SmR: streptomycin-resistant
TEs: transposable elements
