# Supplementary material for: The pQBR mercury resistance plasmids: a model set of sympatric environmental mobile genetic elements
Source: Microb Genom. 2026 Jul 22;12(7):001789. doi: 10.1099/mgen.0.001789 (PMC13391042; doi:10.1099/mgen.0.001789)
Supplement: Supplementary Material 1. [file mgen-12-01789-s001.pdf]

Supplementary Information for

The pQBR mercury resistance plasmids: a model set of sympatric environmental mobile genetic elements

Victoria T. Orr[1](0000-0001-6283-9459), Ellie Harrison[2] (0000-0002-2050-4631), Damian W. Rivett[3] (0000-0002-1852-6137), [4]Rosanna C. T. Wright (0000-0002-8095-8256), James P. J. Hall[1] (0000-0002-4896-4592)

Affiliations: [1]Department of Evolution, Ecology and Behaviour, University of Liverpool, Liverpool UK
[2]School of Biosciences, University of Sheffield, Sheffield UK
[3]Department of Natural Science, Manchester Metropolitan University, Manchester UK
[4]School of Biological Sciences, University of Manchester, Manchester, UK

Correspondence: Victoria Orr (Victoria.Orr@liverpool.ac.uk); James P. J. Hall (j.p.j.hall@liverpool.ac.uk)

Supplementary Methods

Creation of P. fluorescens SBW25 strain contain kanamycin resistant marker on Tn6291

The suicide vector pTS-1_Tn*6291*::KmR was generated from pTS-1 (1) by amplifying 900 bp upstream and downstream fragments homologous to Tn*6291* from *P. fluorescens* SBW25, a 1219 bp fragment containing the *aphA1* gene for kanamycin phosphotransferase and associated promoter region from pUCP18-KmR (2), and the lambda t0 and rrnB T1 terminators from pUC18T-mini-Tn7T-Gm (3) (primer sequences in Table S5). Fragments contained appropriate overhangs for combination into the BamHI/HindIII site of pTS-1 by HiFi assembly (New England Biolabs). Clones were verified by PCR and Sanger sequencing, and the plasmid introduced into *P. fluorescens* SBW25 by electroporation (4). Single crossovers were selected on plates supplemented with tetracycline (100 µg/ml) and tested by colony PCR, before growing and spreading samples on plates supplemented with kanamycin and 10% w/v sucrose to select for second crossovers. The resulting candidate clones were tested for kanamycin resistance, tetracycline susceptibility, and the production of appropriately-sized bands by PCR. The final construct was validated by whole genome sequencing (Illumina 2x250 bp) and found to have incorporated the *aphA1* gene with corresponding promoter and terminator as expected at position 2076740 in the reference sequence AM181176. This location was selected as being downstream of open reading frames on the forward and reverse strands (PFLU_1901, a putative cytochrome D oxidase subunit II, and PFLU_1902, a putative inorganic pyrophosphatase) to minimise potential polar effects. No second-site mutations were detected.

Sequencing and assembly of pQBR plasmids

We used a hybrid method to produce plasmid sequences for subsequent analysis. Whole genome SPAdes assemblies of Illumina reads, viewed in Bandage (0.9.0), resulted in closed contigs for the Group I plasmid pQBR103, the Group IV plasmid pQBR102, and the Group III plasmids pQBR55, pQBR132 and pQBR28. The Group III plasmid pQBR53 could also be resolved from the Illumina assembly owing to the presence of a plasmid backbone with >99.99% nucleotide identity to the known Group III plasmid pQBR55, forming junctions with a contig matching the chromosomal transposon Tn*4652* (also present, but in a different location, in pQBR55). The path of the plasmid backbone and transposon contigs was considered a closed assembly for pQBR53.

ONT sequencing was performed on the following pools (Groups are provided in brackets): pQBR30 (IV), pQBR5 (I) and pQBR24 (II); pQBR57 (IV), pQBR4 (I) and pQBR23

(II); pQBR124 (I), pQBR56 (IV) and pQBR105 (NA); pQBR49 (I) and pQBR150 (IV). Plasmids pQBR51 (IV), pQBR11 (I), pQBR47 (I), pQBR106 (I), pQBR127 (IV), pQBR50 (I), pQBR43 (I), and pQBR26 were each sequenced individually. ONT assemblies, produced by Flye and polished using Medaka, were further polished the corresponding Illumina reads using Polypolish 0.5.0, and direct repeats at the start and end of all assemblies were identified by ccfind (5) and removed as assembly artefacts. Short-read sequencing of UWC1(pQBR127) revealed replicons that both resembled Group I and Group III plasmids, but long-read sequencing showed a distinct Group III replicon and no Group I replicon. We concluded that two plasmids were present in the strain used for Illumina sequencing, of which only one remained for subsequent long-read and phenotypic analysis, which we labelled pQBR127. Sequencing of UWC1(pQBR26) identified two separate plasmid sequences harbouring mercury resistance elements: one sequence had coverage similar to the chromosome and transferred into recipients, whereas the other, which had high identity (>99.99% nucleotide identity) to the other Group II plasmids pQBR23 and pQBR24, was at lower coverage (~0.75x chromosomal coverage) and was not detected to transfer (PCR on transconjugants, n = 8 attempts). We labelled the former plasmid pQBR26. Overall, these approaches produced closed plasmid sequences for 20/28 UWC1(pQBR) strains.

In one case (pQBR11), we could not identify any matching Group I contigs from long-read sequencing, but a resolved sequence could be generated from three contigs of the Illumina assembly, which were oriented with reference to pQBR103 and joined to generate the complete draft. In 4 cases (pQBR43, pQBR47, pQBR50, pQBR106), closed sequences could not be obtained, owing to the presence of long (~40 kb) repeated Tn*6290* transposon sequences in the plasmid and the chromosome that could not be resolved by long-read sequencing. For pQBR47 and pQBR106, the Flye assembly of ONT reads suggested that the plasmids had integrated into the chromosome, possibly through Tn*6290* recombination. The chromosomal region was removed for the subsequent plasmid analyses. For pQBR43, the single putative plasmid contig was bound by Tn*6290*-like sequences at either end, and for pQBR50 the assembly was in 2 contigs, each of which was bordered by Tn*6290* copies. In both of these assemblies, Tn*6290* coverage was higher than plasmid and chromosomal coverage, suggesting the presence of multiple Tn*6290* copies in the genome. Sequences were oriented using EMBOSS to place the first base of the first codon of a putative replicase, identified by querying a BLAST database of putative replicases from previously-sequenced pQBR plasmids, at the first position on the forward strand, prior to annotation using bakta (v1.8.2, full database v.5.0.0).

In three cases (pQBR1, pQBR8, pQBR58), long deep contigs could not be obtained during the initial comparative analysis, and instead short contigs matching only a putative mercury resistance transposon, and/or longer contigs at lower coverage (<0.5x chromosome

coverage) were identified. In these cases, we concluded that the plasmid was at intermediate frequency in the sequenced samples, indicative of plasmid loss from the population following capture of mercury resistance to the chromosome, and attempts to reisolate the plasmid by conjugation into a new host were unsuccessful.

Three plasmids, sequenced in an earlier analysis (6), were sequenced again from the original stock: pQBR55 (Genbank: LN713927.1), pQBR57 (Genbank: LN713926.1) and pQBR103 (AM235768.1). While the resolved sequence of pQBR103 was identical between the original and resequenced samples, pQBR57 and pQBR55 varied in the presence of transposons, with the resequenced pQBR57 harbouring Tn*4652* (bases 136385-153397, similar but not identical to patterns previously observed (7) and the resequenced pQBR55 lacking Tn*4652*. The resequenced assemblies were labelled pQBR57R and pQBR55R. We included in our analysis the published sequence of pQBR44 (Genbank: CDLQ010000001-CDLQ010000002) (6), treating the two contigs as 'drafts', as described above. Throughout, we use bakta (v1.8.2, full database v.5.0.0; metagenome mode) re-annotations of all sequences when referring to gene annotations and locus tags, to keep consistency across the dataset; these are provided on Github (www.github.com/jpjh/PQBR_PLASMIDS).

Measuring plasmid fitness effects

Plasmid-containing SBW25-Tn*6291*::KmR strains to be tested were competed against a reference plasmid-free SBW25::SmR-*lacZ*. A control comparison between plasmid-free SBW25-Tn*6291*::KmR and SBW25::SmR-*lacZ* was conducted to test for any effects of the KmR marker. Single colonies were obtained from each strain and cultured in 5 ml KB broth overnight. The following day, competitions were mixed at approximately 1:1 ratio, diluted 1:100 into fresh KB broth, and incubated for 24 hours. Dilutions of the initial competition mix were plated onto KB agar containing X-gal to obtain start counts. After incubation, competitions were diluted and spread on KB agar containing X-gal to obtain end counts. Competitors could then be distinguished using colour, with SBW25::SmR-*lacZ* appearing blue compared to the white competitor strains.

Plate counts were transformed into competitive fitness value "w" using the following equations (8):

$$m = \ln\left(\frac{N/_{N_0}}{T}\right)$$

$$w = \frac{m_{test}}{m_{reference}}$$

N is the total colonies of a strain at the end of the experiment and N_0 is the total colonies of strain at the start of the experiment. T is the experiment duration, in this case 24 hours.

Supplementary Results and Discussion

Substitutions in Tn5042 across collection and mer operons in pQBR26 and pQBR105

All copies of Tn*5042* within the pQBR collection had 35 nucleotide substitutions (Supplementary: Table S2), 7 insertions and 4 deletions compared with the reference sequence (AJ563380.2) Group I plasmid copies (except pQBR11 and pQBR49) contained an additional G296>T substitution (within *merR*) and pQBR132 contained both a C733>A (intergenic region) and G4635>T (within hypothetical protein).

Tn*5042* was not present in pQBR26 and pQBR105, TnCentral could not identify any complete mercury transposons within these sequences. Plasmid pQBR26 contains a *merRTPCADE* operon as well as separate *merED, merPTR* and *merR* genes and pQBR105 encodes *merR, merRTP* and *merPTR*. These *mer* genes are all flanked by putative transposase genes suggesting they are likewise present on TEs.

Additional details of Tn4652 and Tn6290 gene content and putative Group II transposon

Tn*4652* harbours a metal-resistant determinant, *mrdH*, that confers cadmium and zinc resistance in *P. putida* KT2440, and *mreA*, a metal-sensing transcriptional repressor which was shown to be conserved in association with *mrdH* (9). Annotation for bakta suggests the Tn*6290* sequence encodes copper resistance genes, putative copper efflux transporters as well as other heavy metal transportation genes. Tn*6291* contains many hypothetical proteins but also a putative *prtN* gene, which in *Pseudomonas aeruginosa* is involved in activation of pyocin production (10). Besides the full-length TEs described in the main text, by comparing sequences with those in TnCentral we also found some matches to variants of Tn*4662* amongst the Group II plasmids, which may contain a toxin-antitoxin system (11).

Putative transposon transfer mechanisms

Bakta annotation of the Tn*6290* sequence (BK010246.1) revealed elements of a Tn*7* family associated transposition system (TnsC, TnsD), 2 transposases of unassigned family and an inner IS*66* insertion sequence transposase and associated protein. Tn*7* family transposable elements can undergo both cut-paste and copy-paste activity, influenced by the *tnsA* gene (12); given the high coverage of Tn*6290* in our data it is likely that it has utilised a copy-paste mechanism.

Bakta annotation of Tn*6291*, revealed the presence of an IS*21*-like transposase and helper ATPase (IstA and IstB; PFLU_1887 and PFLU_1888) suggesting a copy-paste transposition mechanism (13). Given we found copies of Tn*6291* on both plasmid and host chromosome, this further supports a copy-paste mechanism.

A previous analysis of Tn*4652* suggested its transposase gene was located at one end, resembling the Tn*3* family of transposons that utilise a copy-paste transposition mechanism (14). Further work was done sequencing the Tn*3* transposase and related promotor at the beginning and end of the sequence, which our Tn*4652* matches as well as the reference sequence AF151431.1 (15). However, Tn*4652* (AF151431.1) also contains various putative transposase genes in the middle of the sequence, suggesting a composite element, which is unclear whether this was present in the Tn*4652* initially studied (14,15). Present are transposases of the Tn*3*-family IS*5*-family, IS*3*-family and a DDE transposase, as well as a Tn*3* regulator/resolvase (*tnpC*). Similar to Tn*4651*, of which Tn*4652* is a deletion derivative, it contains a TnpT protein, associated with cointegrate-resolution, usually alongside a tyrosine recombinase TnpS (16). Tn*4652* has no TnpS labelled by bakta but a Tyr recombinase domain-containing protein is adjacent to the TnpT. Taken together, it is likely that Tn*4562* uses a copy-paste style mechanism.

Mercury transposon Tn*5042* is described as a IS*66*-related transposon, suggesting a cut-paste mechanism of transposition (17), though previous studies have indicated that Tn*5042* can amplify to many copies in a lineage suggesting that an alternative replicative route may exist (7). Bakta annotation confirmed the presence of a TnpA and TnpB protein, as well as a IS*66*-family transposase associated with this mechanism of transposition.

Gene content variations in the Group IV plasmids pQBR30 and pQBR102

Plasmid pQBR30 lacked several ‘phage-related’ genes present in other Group IV plasmids, while pQBR102 did not encode a ferredoxin-NADP+ reductase, homologues of which are involved in redox metabolism (18). Several genes within pQBR30 and pQBR102 were truncated, including a HNH endonuclease in pQBR102 (pQBR102_01690). Unique to

pQBR30 and pQBR102 is an autotransporter outer membrane beta-barrel domain-containing protein (PQBR102_01340, PQBR30_01205).

Plasmid transfer of chromosomal traits varies across pQBR plasmids, and is not determined by conjugation rate – comparison of ratios

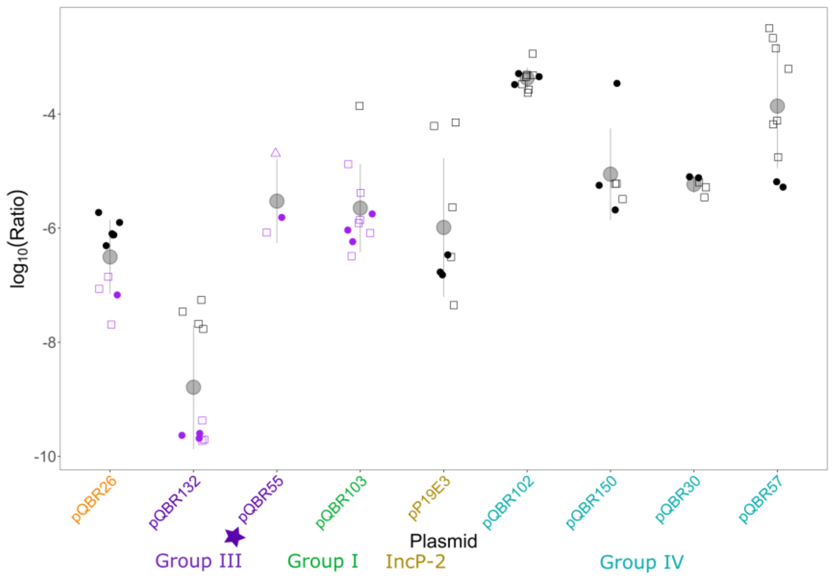

Figure S1 Conjugation rate is not a good predictor of transposon transfer. Ratios calculated by dividing plasmid transposon transfer rate by plasmid conjugation rate, which were used for analysis. This figure represents a summary of two experiments performed a month apart, with 2-3 different donor transconjugant strains for each plasmid identified by shape. Large points represent average measurement. Purple points indicate where transposon transfer measurement was below limit of detection for experiment and given an arbitrary small value. Colours of plasmid labels represents the group the plasmid belongs to, labelled beneath. Star (pQBR55) indicates that data for this plasmid was collected separately from the rest of the dataset under similar experimental conditions.

Comparative fitness of pQBR plasmids

To investigate whether these patterns could be explained by the effect of the plasmids on cell fitness, we conducted growth curve and competitive fitness assays. We focused on the Group IV plasmids, owing to their significant within-group variation in transposon transfer rates.

Plasmid-carrying strains suffered a decrease in maximum per capita growth rate compared with a plasmid-free control (ANOVA: $F_{6,35} = 8.355$, $p < 0.01$) (Figure S2A). Using head-to-head competitive fitness assays, we detected differences in relative fitness depending on which plasmid was being carried ($F_{10,129} = 24.23$, $p < 0.01$). We did not detect any differences in fitness costs of the Group IV plasmids, except a small difference between

pQBR102 and pQBR57 ($p = 0.04$) (Figure S2B), though fitness costs relative to a plasmid-free control were also not detected, possibly owing to high conjugation rates obscuring differences in this assay. Based on TukeyHSD post hoc tests, intergroup differences were observed between pQBR103 (Group I) and all other groups ($p < 0.01$), and Group III plasmid pQBR132 had a higher relative fitness than all plasmids in other groups, except Group IV plasmids pQBR102 and pQBR30 (all $p < 0.02$). There was no obvious correlation between plasmid fitness effects and the efficiency of the plasmid in transfer of a chromosomal locus, suggesting that the striking differences in transposon acquisition and transfer are due to more subtle differences in plasmid activity or gene content.

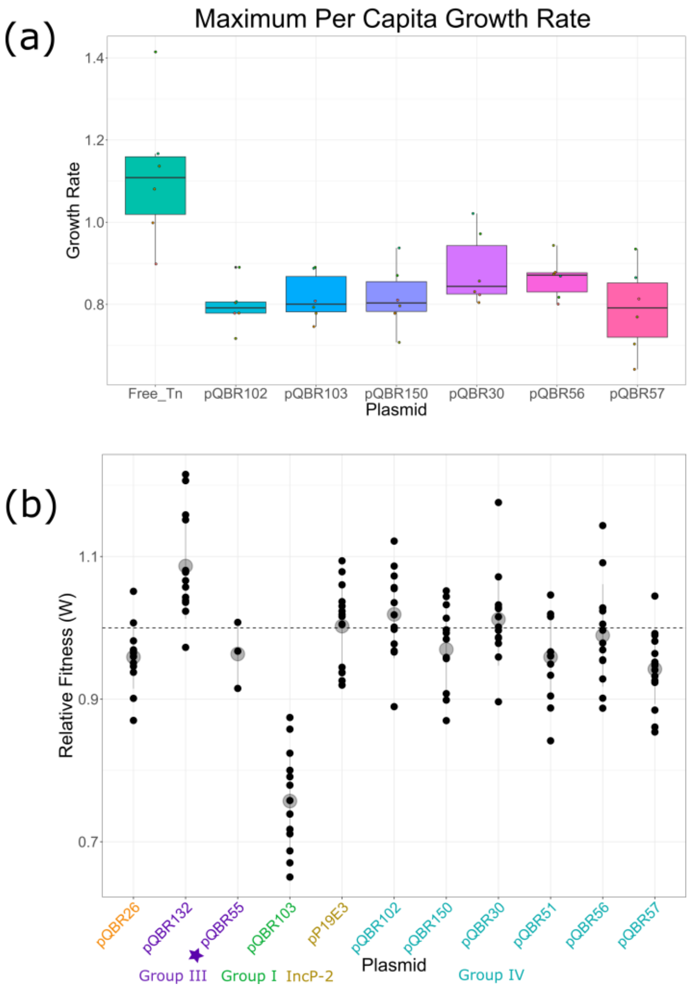

Figure S2 A. Maximum per capita growth rate was reduced in plasmid carrying strains compared to a plasmid free strain. **B.** Intergroup plasmid fitness costs were greater than intra-group differences. Group IV plasmid fitness cost did not vary significantly, except a small difference between pQBR102 and pQBR57. Colours of plasmid labels represents the group the plasmid belongs to, labelled beneath. Star (pQBR55) indicates that data for this plasmid was collected separately from the rest of the dataset under similar experimental conditions.

References

1. Campilongo R, Fung RKY, Little RH, Grenga L, Trampari E, Pepe S, et al. One ligand, two regulators and three binding sites: How KDPG controls primary carbon metabolism in Pseudomonas. PLOS Genetics. 2017 Jun 28;13(6):e1006839. doi:10.1371/journal.pgen.1006839

2. Schweizer HP. Escherichia-Pseudomonas shuttle vectors derived from pUC18/19. Gene. 1991 Jan 2;97(1):109–21. doi:10.1016/0378-1119(91)90016-5 PubMed PMID: 1899844.

3. Choi KH, Gaynor JB, White KG, Lopez C, Bosio CM, Karkhoff-Schweizer RR, et al. A Tn7-based broad-range bacterial cloning and expression system. Nat Methods. 2005 Jun;2(6):443–8. doi:10.1038/nmeth765

4. Choi KH, Ayush K, Schweizer HP. A 10-min method for preparation of highly electrocompetent Pseudomonas aeruginosa cells: Application for DNA fragment transfer between chromosomes and plasmid transformation. Journal of Microbiological Methods. 2006 Mar 1;64(3):391–7. doi:10.1016/j.mimet.2005.06.001

5. Nishimura Y. yosuken/ccfind [Ruby]. 2024.Available from: https://github.com/yosuken/ccfind

6. Hall JPJ, Harrison E, Lilley AK, Paterson S, Spiers AJ, Brockhurst MA. Environmentally co-occurring mercury resistance plasmids are genetically and phenotypically diverse and confer variable context-dependent fitness effects. Environ Microbiol. 2015 Dec;17(12):5008–22. doi:10.1111/1462-2920.12901 PubMed PMID: 25969927; PubMed Central PMCID: PMC4989453.

7. Hall JPJ, Williams D, Paterson S, Harrison E, Brockhurst MA. Positive selection inhibits gene mobilization and transfer in soil bacterial communities. Nat Ecol Evol. 2017 Sep;1(9):1348–53. doi:10.1038/s41559-017-0250-3

8. Lenski RE. Quantifying fitness and gene stability in microorganisms. Biotechnology. 1991;15:173–92. doi:10.1016/b978-0-409-90199-3.50015-2 PubMed PMID: 2009380.

9. Haritha A, Sagar KP, Tiwari A, Kiranmayi P, Rodrigue A, Mohan PM, et al. MrdH, a Novel Metal Resistance Determinant of Pseudomonas putida KT2440, Is Flanked by Metal-Inducible Mobile Genetic Elements. J Bacteriol. 2009 Oct;191(19):5976–87. doi:10.1128/JB.00465-09 PubMed PMID: 19648243; PubMed Central PMCID: PMC2747888.

10. Matsui H, Sano Y, Ishihara H, Shinomiya T. Regulation of pyocin genes in Pseudomonas aeruginosa by positive (prtN) and negative (prtR) regulatory genes. J Bacteriol. 1993 Mar 1;175(5):1257–63. doi:10.1128/jb.175.5.1257-1263.1993 PubMed PMID: 8444788; PubMed Central PMCID: PMC193209.

11. Yano H, Miyakoshi M, Ohshima K, Tabata M, Nagata Y, Hattori M, et al. Complete Nucleotide Sequence of TOL Plasmid pDK1 Provides Evidence for Evolutionary History of IncP-7 Catabolic Plasmids. J Bacteriol. 2010 Sep;192(17):4337–47. doi:10.1128/JB.00359-10 PubMed PMID: 20581207; PubMed Central PMCID: PMC2937381.

12. May EW, Craig NL. Switching from Cut-and-Paste to Replicative Tn7 Transposition. Science. 1996 Apr 19;272(5260):401–4. doi:10.1126/science.272.5260.401

13. Berger B, Haas D. Transposase and cointegrase: specialized transposition proteins of the bacterial insertion sequence IS21 and related elements. Cell Mol Life Sci. 2001 Mar;58(3):403–19. doi:10.1007/PL00000866 PubMed PMID: 11315188; PubMed Central PMCID: PMC11337337.

14. Tsuda M, Iino T. Genetic analysis of a transposon carrying toluene degrading genes on a TOL plasmid pWWO. Molec Gen Genet. 1987 Dec 1;210(2):270–6. doi:10.1007/BF00325693

15. Hõrak R, Kivisaar M. Expression of the Transposase Gene tnpA of Tn4652 Is Positively Affected by Integration Host Factor. J Bacteriol. 1998 Jun;180(11):2822–9. doi:10.1128/jb.180.11.2822-2829.1998 PubMed PMID: 9603867; PubMed Central PMCID: PMC107244.

16. Yano H, Genka H, Ohtsubo Y, Nagata Y, Top EM, Tsuda M. Cointegrate-resolution of toluene-catabolic transposon Tn*4651*: Determination of crossover site and the segment required for full resolution activity. Plasmid. 2013 Jan 1;69(1):24–35. doi:10.1016/j.plasmid.2012.07.004

17. Mindlin S, Minakhin L, Petrova M, Kholodii G, Minakhina S, Gorlenko Z, et al. Present-day mercury resistance transposons are common in bacteria preserved in permafrost grounds since the Upper Pleistocene. Research in Microbiology. 2005 Dec 1;156(10):994–1004. doi:10.1016/j.resmic.2005.05.011

18. Monchietti P, López Rivero AS, Ceccarelli EA, Catalano-Dupuy DL. A new catalytic mechanism of bacterial ferredoxin-NADP+ reductases due to a particular NADP+ binding mode. Protein Sci. 2021 Oct;30(10):2106–20. doi:10.1002/pro.4166 PubMed PMID: 34382711; PubMed Central PMCID: PMC8442965.
